# Supplementary material for: KLF5 controls subtype-independent highly interactive enhancers in pancreatic cancer to regulate cell survival
Source: Sci Adv. 2026 Mar 18;12(12):eaea2106. doi: 10.1126/sciadv.aea2106 (PMC12998525; doi:10.1126/sciadv.aea2106)

Science Advances
AAAS

# Supplementary Materials for

## KLF5 controls subtype-independent highly interactive enhancers in pancreatic cancer to regulate cell survival

Thomas L. Ekstrom *et al.*

Corresponding author: Steven A. Johnsen, steven.johnsen@uni-tuebingen.de

*Sci. Adv.* **12**, eaea2106 (2026)
DOI: 10.1126/sciadv.aea2106

**This PDF file includes:**

Figs. S1 to S25
Tables S1 and S2
Western blot images

fig. S1

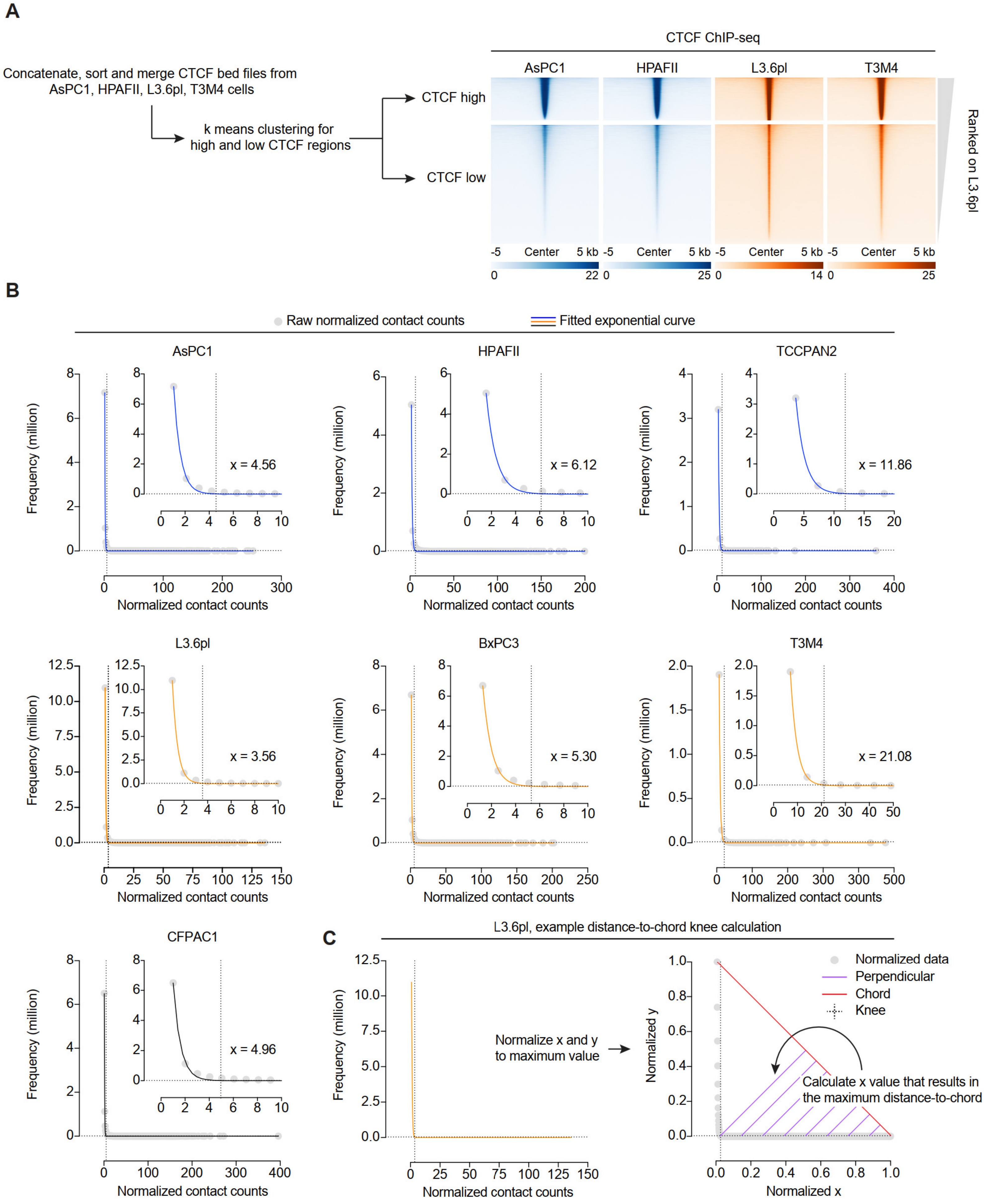
A
Concatenate, sort and merge CTCF bed files from AsPC1, HPAFII, L3.6pl, T3M4 cells
k means clustering for high and low CTCF regions
CTCF high
CTCF low
CTCF ChIP-seq
AsPC1
HPAFII
L3.6pl
T3M4
Ranked on L3.6pl
-5
Center
5 kb
B
Raw normalized contact counts
Fitted exponential curve
AsPC1
HPAFII
TCCPAN2
L3.6pl
BxPC3
T3M4
CFPAC1
Frequency (million)
Normalized contact counts
x = 4.56
x = 6.12
x = 11.86
x = 3.56
x = 5.30
x = 21.08
x = 4.96
C
L3.6pl, example distance-to-chord knee calculation
Normalize x and y to maximum value
Normalized y
Normalized x
Normalized data
Perpendicular
Chord
Knee
Calculate x value that results in the maximum distance-to-chord

## fig. S1

**fig. S1. Identification of subtype-independent highly interactive enhancers**.

(**A**) Schematic and heatmap of k means clustering of all CTCF regions in AsPC1, HPAFII, L3.6pl and T3M4 cells. (**B**) Ranked normalized contact counts and fitted exponential curve of H3K4me3-anchored HiChIP data in classical (AsPC1, HPAFII, TCCPAN2), basal-like (BxPC3, L3.6pl, T3M4) and intermediate (CFPAC1) cells. Cross section represents the knee point. Contact counts were normalized to the cell line with the highest valid_interaction_rmdup. (**C**) Example calculation of the distance-to-chord method to calculate the knee point for each cell line.

fig. S2

A

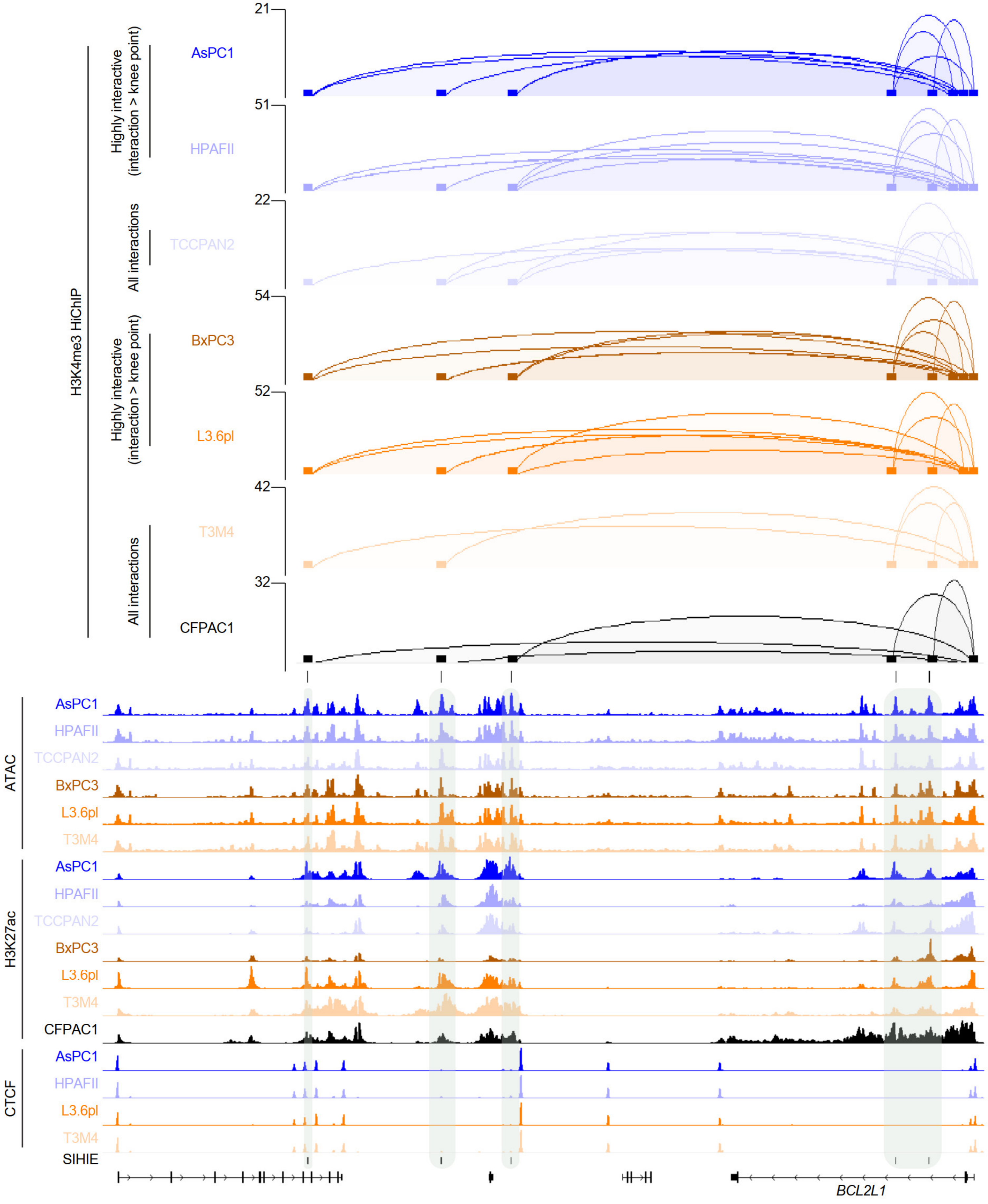

H3K4me3 HiChIP
Highly interactive (interaction > knee point)
All interactions
Highly interactive (interaction > knee point)
All interactions
AsPC1
HPAFII
TCCPAN2
BxPC3
L3.6pl
T3M4
CFPAC1
21
51
22
54
52
42
32
ATAC
H3K27ac
CTCF
SIHIE
BCL2L1

## fig. S2

**fig. S2. Example subtype-independent highly interactive enhancer loci.**

(**A**) Example integrated genome viewer (IGV) track of multiple subtype-independent highly interactive enhancers (SIHIE) across all cell systems interacting with an essential gene, *BCL2L1*. These loci are positive for ATAC, H3K27ac and are lowly bound by CTCF across classical (AsPC1, HPAFII, TCCPAN2), basal-like (BxPC3, L3.6pl, T3M4) cells.

**fig. S3**

**A**

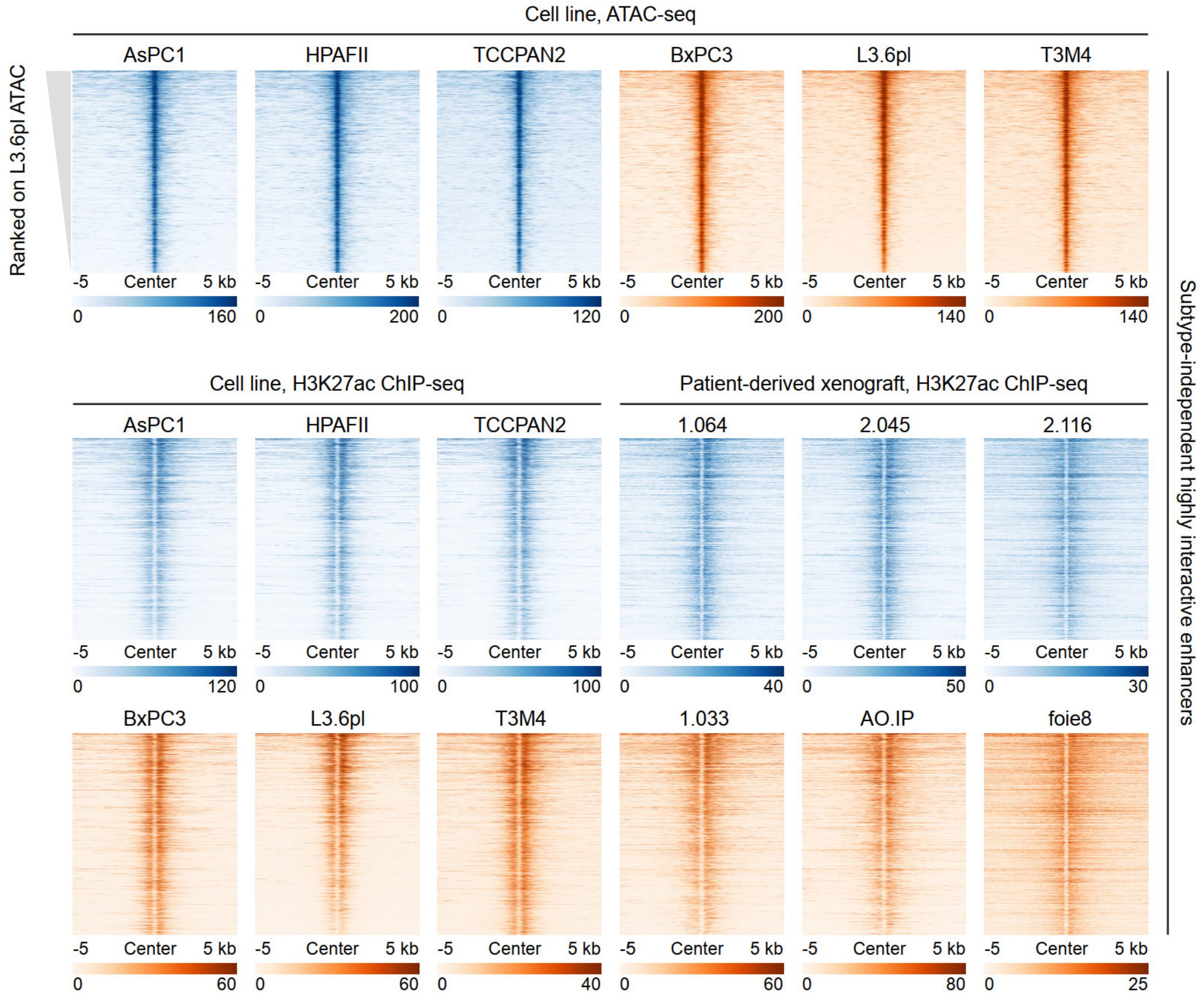


**B**

HOMER known motif analysis, p ≤ 1e-100 and target sequences with motif ≥ 25%

| Name | Motif | p-val | % of target seq with motif | Name | Motif | p-val | % of target seq with motif |
|---|---|---|---|---|---|---|---|
| Basic leucine zipper | | | | Zinc finger | | | |
| FOS | | 1e-927 | 55.31 | KLF5 | | 1e-112 | 40.19 |
| | | | | ETS | | | |
| FRA1 | | 1e-923 | 54.49 | | | | |
| FRA2 | | 1e-894 | 51.69 | ETS1 | | 1e-120 | 27.94 |
| ATF3 | | 1e-883 | 56.61 | FLI1 | | 1e-115 | 28.62 |
| JUNB | | 1e-862 | 52.99 | ERG | | 1e-115 | 37.07 |
| BATF | | 1e-862 | 55.60 | ETV2 | | 1e-103 | 25.19 |
| FOSL2 | | 1e-857 | 45.51 | ETV1 | | 1e-102 | 32.05 |
| AP-1 | | 1e-833 | 57.53 | | | | |
| JUN-AP1 | | 1e-769 | 38.85 | | | | |

## fig. S3

**fig. S3. Subtype-independent highly interactive enhancers are active across cell lines and PDX models.**

(**A**) Heatmap of chromatin accessibility and H3K27ac in multiple classical and basal-like A cell lines and patient-derived xenografts on subtype-independent highly interactive enhancers. Ranked descending on ATAC-seq signal in L3.6pl cells. (**B**) HOMER motif analysis results on the top motifs enriched in subtype-independent highly interactive enhancers.

fig. S4

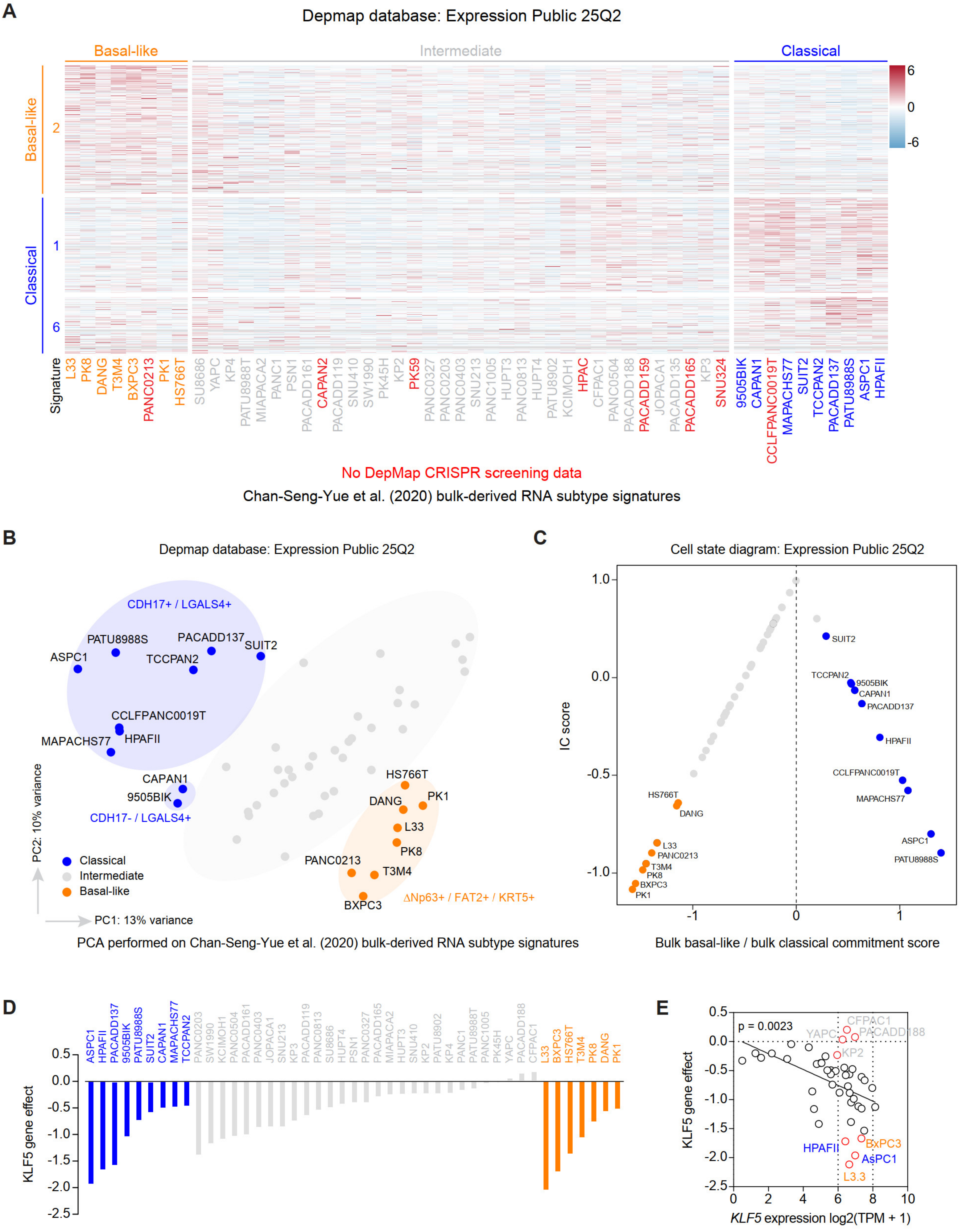
A
Depmap database: Expression Public 25Q2
Basal-like
Intermediate
Classical
Basal-like
Classical
2
1
6
6
0
-6
Signature
L33 PK8 DANG T3M4 BXPC3 PANC0213 PK1 HS766T
SU8686 YAPC KP4 PATU8988T MIAPACA2 PANC1 PSN1 PACADD161 CAPAN2 PACADD119 SNU410 SW1990 PK45H KP2 PK59 PANC0327 PANC0203 PANC0403 SNU213 PANC1005 HUPT3 PANC0813 HUPT4 PATU8902 KCIMOH1 HPAC CFPAC1 PANC0504 PACADD188 PACADD159 JOPACA1 PACADD135 PACADD165 KP3 SNU324
9505BIK CAPAN1 CCLFPANC0019T MAPACHS77 SUIT2 TCCPAN2 PACADD137 PATU8988S ASPC1 HPAFII
No DepMap CRISPR screening data
Chan-Seng-Yue et al. (2020) bulk-derived RNA subtype signatures
B
Depmap database: Expression Public 25Q2
CDH17+ / LGALS4+
PATU8988S
PACADD137
SUIT2
ASPC1
TCCPAN2
CCLFPANC0019T
MAPACHS77
HPAFII
CAPAN1
9505BIK
CDH17- / LGALS4+
HS766T
DANG
PK1
L33
PK8
PANC0213
T3M4
BXPC3
ΔNp63+ / FAT2+ / KRT5+
Classical
Intermediate
Basal-like
PC2: 10% variance
PC1: 13% variance
PCA performed on Chan-Seng-Yue et al. (2020) bulk-derived RNA subtype signatures
C
Cell state diagram: Expression Public 25Q2
IC score
1.0
0.0
-0.5
-1.0
-1
0
1
SUIT2
TCCPAN2
9505BIK
CAPAN1
PACADD137
HPAFII
CCLFPANC0019T
MAPACHS77
ASPC1
PATU8988S
HS766T
DANG
L33
PANC0213
T3M4
PK8
BXPC3
PK1
Bulk basal-like / bulk classical commitment score
D
KLF5 gene effect
0.5
0.0
-0.5
-1.0
-1.5
-2.0
-2.5
ASPC1 HPAFII PACADD137 9505BIK PATU8988S SUIT2 CAPAN1 MAPACHS77 TCCPAN2
PANC0203 SW1990 KCIMOH1 PANC0504 PANC0403 PACADD161 JOPACA1 SNU213 KP3 PACADD119 PANC0813 SU8686 HUPT4 PSN1 PANC0327 PACADD165 MIAPACA2 HUPT3 SNU410 KP2 PATU8902 KP4 PANC1 PATU8988T PANC1005 PK45H YAPC PACADD188 CFPAC1
L33 BXPC3 HS766T T3M4 PK8 DANG PK1
E
p = 0.0023
CFPAC1
YAPC
PACADD188
KP2
HPAFII
BxPC3
AsPC1
L3.3
KLF5 gene effect
0.5
0.0
-0.5
-1.0
-1.5
-2.0
-2.5
0
2
4
6
8
10
KLF5 expression log2(TPM + 1)

## fig. S4

**fig. S4. Stratification of molecular subtypes in pancreatic cancer cell lines.**

(**A**) Clustering of pancreatic cancer cell lines from the DepMap database using the gene signatures 1, 2 and 6 from Chan-Seng-Yue et al. The heatmap shows the raw z-score values generated on transcript per million (TPM) values from RNA-seq. (**B**) PCA of cell lines using the gene signatures 1, 2 and 6 from Chan-Seng-Yue et al. Blue dots depict classical samples, grey dots depict intermediate samples, orange dots depict basal-like samples. (**C**) Bulk-derived cell state diagram for pancreatic cancer cell lines. Blue dots depict classical samples, grey dots depict intermediate samples, orange dots depict basal-like samples. (**D**) Ranking of KLF5 gene effect per cell line for classical (blue), intermediate (grey), and basal-like cell lines (orange) from the DepMap database. (**E**) Correlation between *KLF5* gene expression (x-axis) and *KLF5* gene effect from CRISPR-dependency data (y-axis) from the DepMap database.

fig. S5

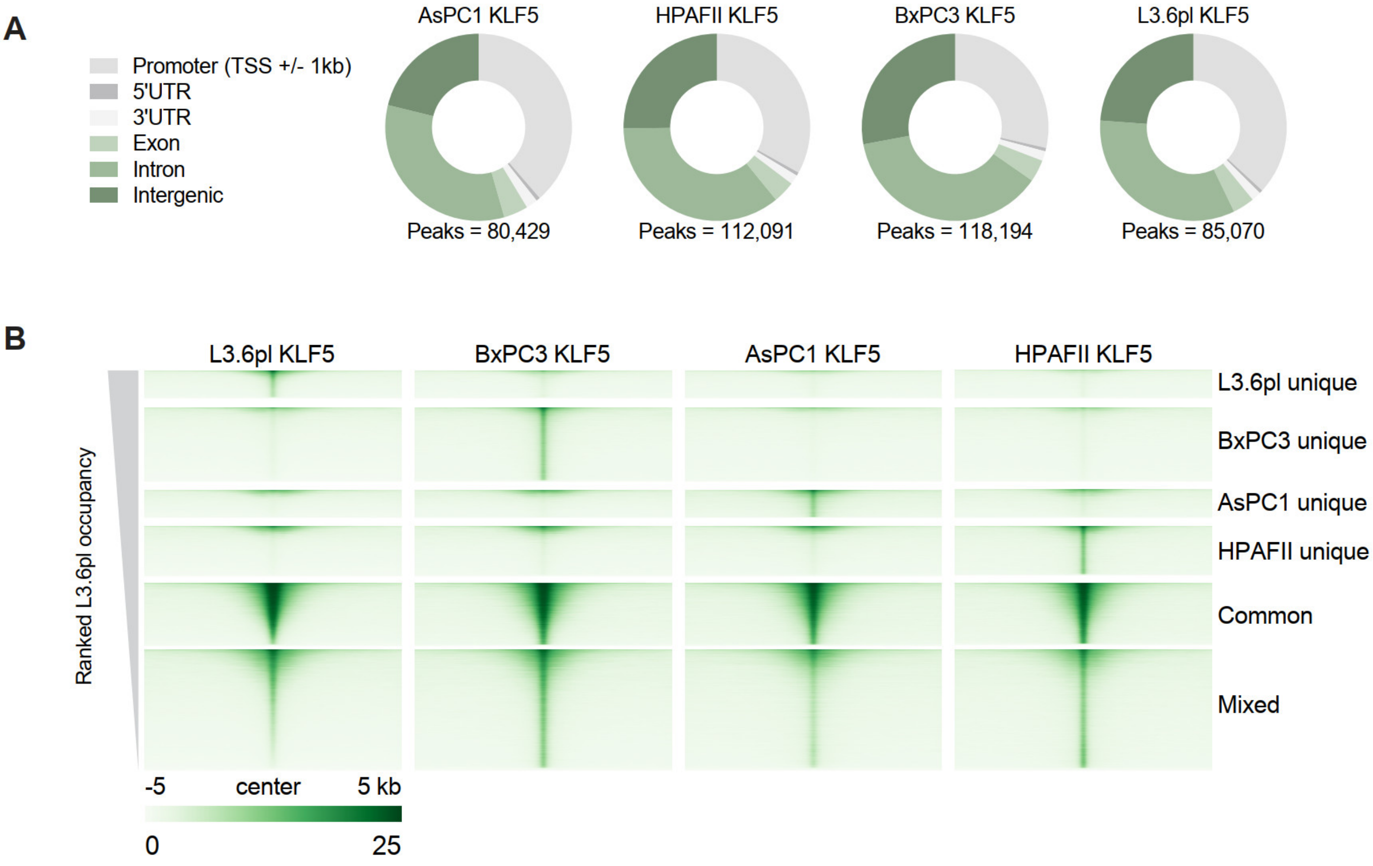


C

| | KLF5 *de novo* motif | P-val | Rank | KLF5 known motif | P-val | Rank |
|---|---|---|---|---|---|---|
| AsPC1 unique | KLF5 motif not detected | | | 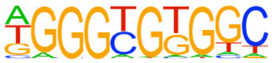 | $1 \times -10^{919}$ | #1 |
| HPAFII unique | 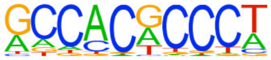 | $1 \times -10^{2255}$ | #1 | | $1 \times -10^{2119}$ | #2 |
| BxPC3 unique | 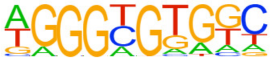 | $1 \times -10^{2775}$ | #1 | | $1 \times -10^{2634}$ | #1 |
| L3.6pl unique | KLF5 motif not detected | | | | $1 \times -10^{433}$ | #3 |
| Common | 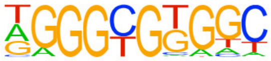 | $1 \times -10^{4067}$ | #1 | | $1 \times -10^{3824}$ | #1 |
| Mixed | 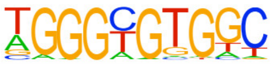 | $1 \times -10^{6320}$ | #1 | | $1 \times -10^{6502}$ | #1 |

**fig. S5. KLF5 primarily binds distal regulatory elements.**

(**A**) ChIPseeker analysis showing genome-wide annotation of KLF5 peaks in AsPC1, HPAFII, BxPC3 and L3.6pl cell lines. (**B**) Heatmap of KLF5 binding sites that are unique to each cell line, common (present in all four cell lines) or mixed (present in two or more but not all four cell lines). Ranked descending on KLF5 signal in L3.6pl cells. (**C**) HOMER motif analysis results on the regions described in (B).

**fig. S6**

**A**

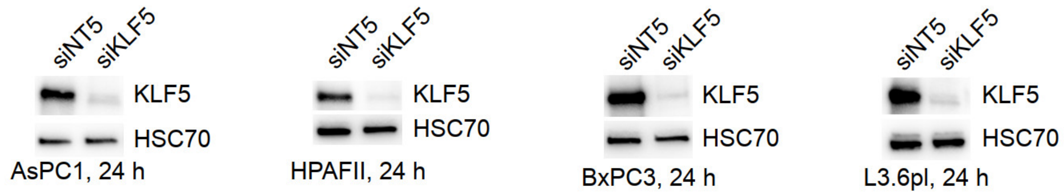


**B**

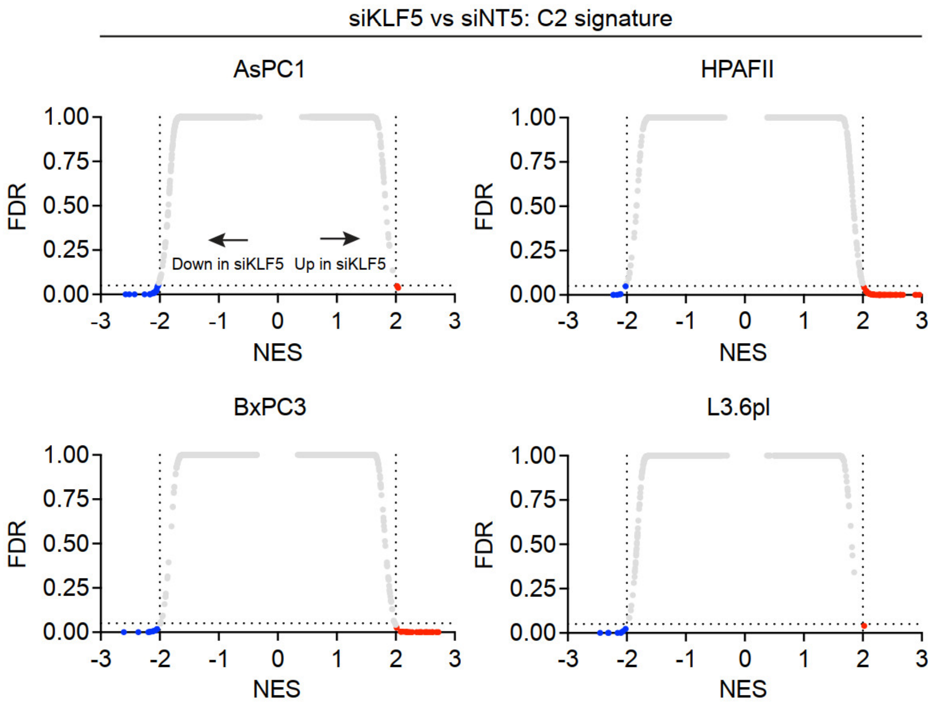


| Common | AsPC1 | HPAFII | BxPC3 | L3.6pl |
|---|---|---|---|---|
| Anderson cholangiocarcinoma class 2 | -2.57 | -2.57 | -2.00 | -2.45 |
| Reactome keratinization | -2.11 | -2.07 | -2.09 | -2.15 |
| Reactome of the cornified envelope | -2.16 | -2.15 | -2.22 | -2.09 |
| Cromer metastasis down | -2.51 | -2.02 | -2.36 | -2.02 |

**C**

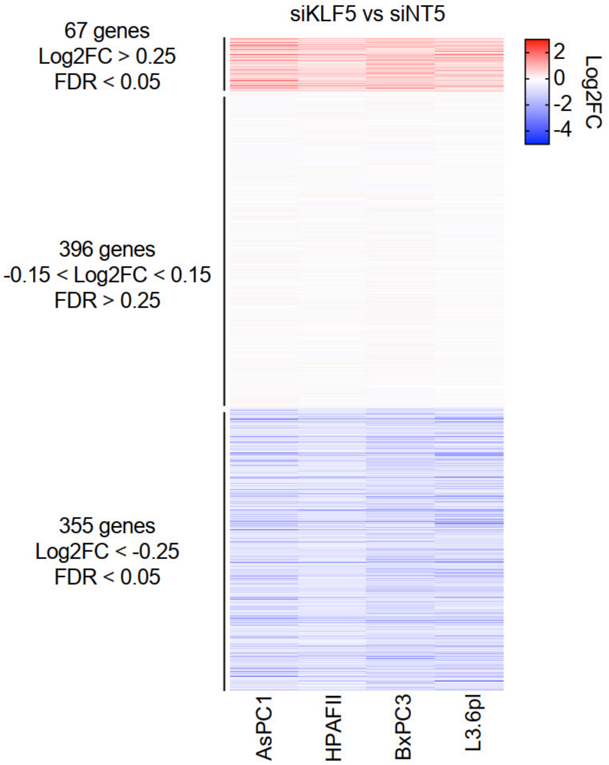


**fig. S6. Transcriptomic analysis upon KLF5 depletion.**

(**A**) Representative Western blot analysis of KLF5 after 24 h KLF5 knockdown in AsPC1, HPAFII, BxPC3 and L3.6pl cells. HSC70 was used as a loading control. (**B**) Gene Set Enrichment Analysis (GSEA) after 24 h KLF5 knockdown in AsPC1, HPAFII, BxPC3 and L3.6pl cells. Normalized enrichment score (NES) is on x-axis and false discovery rate (FDR) on y-axis. Common significantly downregulated pathways are displayed on the table. n = 3 biological replicates. (**C**) Heatmap of Log2FC values of commonly up-, un- and downregulated genes across AsPC1, HPAFII, BxPC3 and L3.6pl cells.

fig. S7

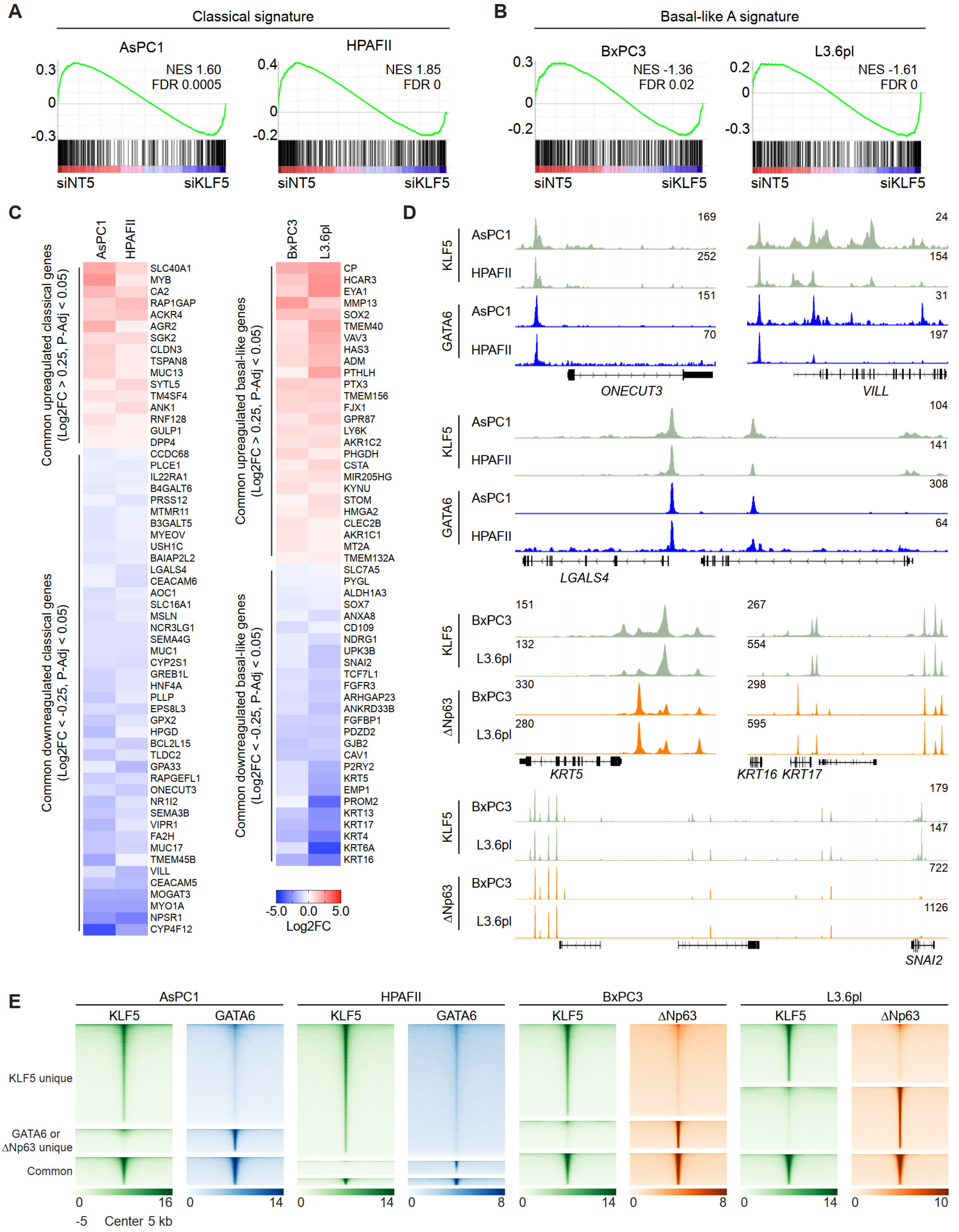

A
Classical signature
AsPC1
NES 1.60
FDR 0.0005
HPAFII
NES 1.85
FDR 0
siNT5
siKLF5
B
Basal-like A signature
BxPC3
NES -1.36
FDR 0.02
L3.6pl
NES -1.61
FDR 0
C
AsPC1
HPAFII
Common upreagulated classical genes (Log2FC > 0.25, P-Adj < 0.05)
SLC40A1
MYB
CA2
RAP1GAP
ACKR4
AGR2
SGK2
CLDN3
TSPAN8
MUC13
SYTL5
TM4SF4
ANK1
RNF128
GULP1
DPP4
Common downreagulated classical genes (Log2FC < -0.25, P-Adj < 0.05)
CCDC68
PLCE1
IL22RA1
B4GALT6
PRSS12
MTMR11
B3GALT5
MYEOV
USH1C
BAIAP2L2
LGALS4
CEACAM6
AOC1
SLC16A1
MSLN
NCR3LG1
SEMA4G
MUC1
CYP2S1
GREB1L
HNF4A
PLLP
EPS8L3
GPX2
HPGD
BCL2L15
TLDC2
GPA33
RAPGEFL1
ONECUT3
NR1I2
SEMA3B
VIPR1
FA2H
MUC17
TMEM45B
VILL
CEACAM5
MOGAT3
MYO1A
NPSR1
CYP4F12
BxPC3
L3.6pl
Common upreagulated basal-like genes (Log2FC > 0.25, P-Adj < 0.05)
CP
HCAR3
EYA1
MMP13
SOX2
TMEM40
VAV3
HAS3
ADM
PTHLH
PTX3
TMEM156
FJX1
GPR87
LY6K
AKR1C2
PHGDH
CSTA
MIR205HG
KYNU
STOM
HMGA2
CLEC2B
AKR1C1
MT2A
TMEM132A
Common downreagulated basal-like genes (Log2FC < -0.25, P-Adj < 0.05)
SLC7A5
PYGL
ALDH1A3
SOX7
ANXA8
CD109
NDRG1
UPK3B
SNAI2
TCF7L1
FGFR3
ARHGAP23
ANKRD33B
FGFBP1
PDZD2
GJB2
CAV1
P2RY2
KRT5
EMP1
PROM2
KRT13
KRT17
KRT4
KRT6A
KRT16
-5.0 0.0 5.0
Log2FC
D
KLF5
GATA6
AsPC1
HPAFII
169
252
151
70
ONECUT3
24
154
31
197
VILL
104
141
308
64
LGALS4
KLF5
ΔNp63
BxPC3
L3.6pl
151
132
330
280
KRT5
267
554
298
595
KRT16 KRT17
179
147
722
1126
SNAI2
E
AsPC1
HPAFII
BxPC3
L3.6pl
KLF5
GATA6
ΔNp63
KLF5 unique
GATA6 or ΔNp63 unique
Common
0 16
0 14
0 14
0 8
0 14
0 8
0 14
0 10
-5 Center 5 kb

## fig. S7

### fig. S7. KLF5 does not alter subtype identity in pancreatic cancer.

(**A and B**) Gene set enrichment analysis (GSEA) of classical signature (signatures 1 and 6) or basal-like A signature (signature 2) from Chan-Seng-Yue et al. in AsPC1 and HPAFII (A) or BxPC3 and L3.6pl (B) cells, respectively. (**C**) Heatmap of Log2FC values of commonly up- and downregulated classical genes in AsPC1 and HPAFII cells or basal-like A genes in BxPC3 and L3.6pl cells. (**D**) Integrated genome viewer (IGV) track of KLF5 and GATA6 in classical cells or KLF5 and ΔNp63 in basal-like cells at KLF5-dependent classical or basal-like signature genes, respectively. (**E**) Heatmap of KLF5 and GATA6 or ΔNp63 at distal KLF5 unique, GATA6 or ΔNp63 unique and common regions in classical or basal-like cells, respectively.

fig. S8

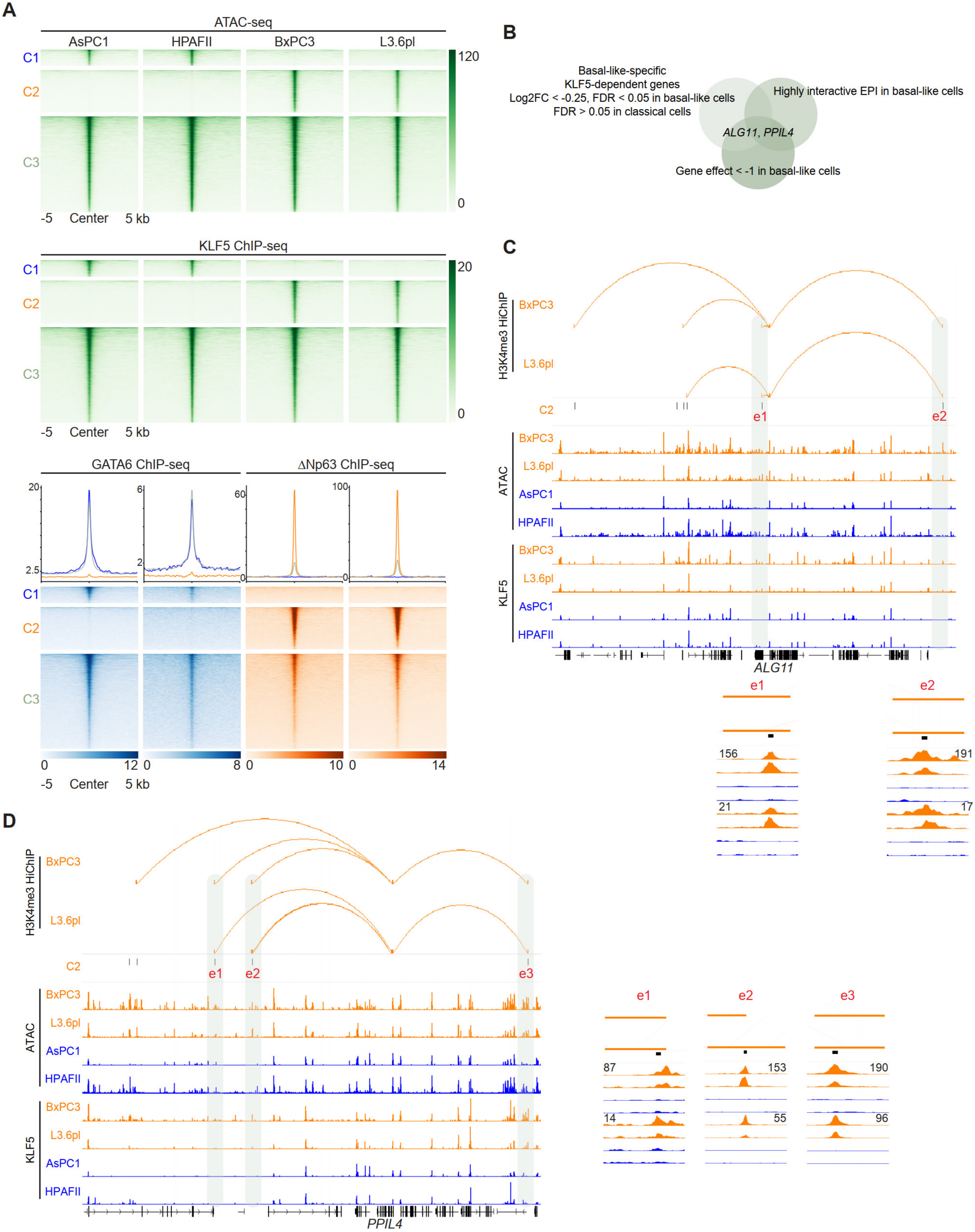

A
ATAC-seq
AsPC1
HPAFII
BxPC3
L3.6pl
C1
C2
C3
120
0
-5
Center
5 kb
KLF5 ChIP-seq
20
GATA6 ChIP-seq
ΔNp63 ChIP-seq
12
8
10
14
B
Basal-like-specific
KLF5-dependent genes
Log2FC < -0.25, FDR < 0.05 in basal-like cells
FDR > 0.05 in classical cells
Highly interactive EPI in basal-like cells
ALG11, PPIL4
Gene effect < -1 in basal-like cells
C
H3K4me3 HiChIP
ATAC
KLF5
e1
e2
ALG11
D
e3
PPIL4

## fig. S8

**fig. S8. KLF5 regulates subtype-dependent and independent gene sets.**

(**A**) Heatmap of open chromatin, KLF5, GATA6 or ΔNp63 at distal ATAC+/KLF5+ regions unique in classical cells (C1), basal-like cells (C2) and common regions (C3). (**B**) Overlap of basal-like-specific KLF5-dependent essential genes in basal-like cell lines that connect highly to a C2 enhancer. (**C and D**) Integrated genome viewer (IGV) tracks of *ALG11* (C) and *PPIL4* (D) interacting with C2 regions.

fig. S9

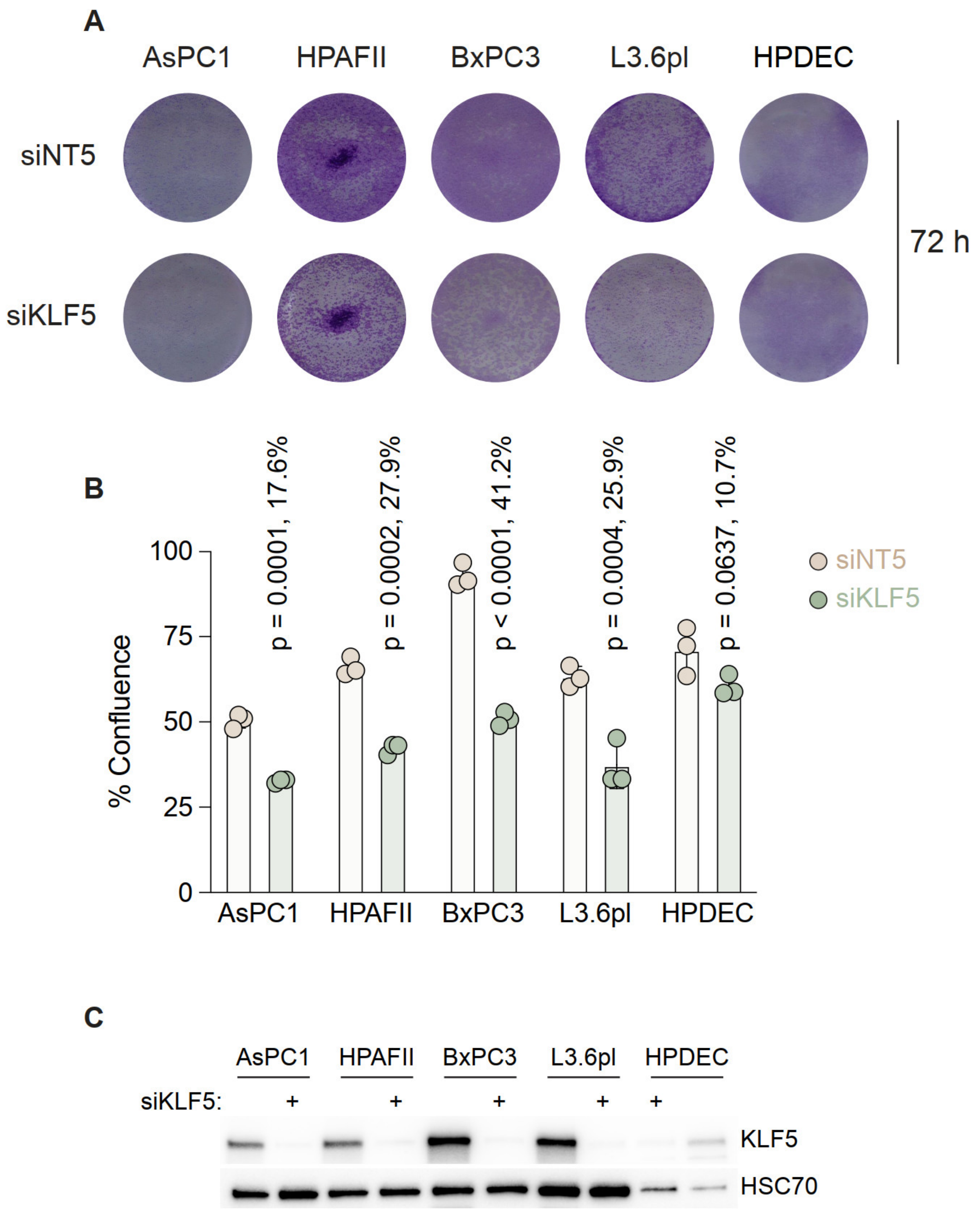


**fig. S9. KLF5 depletion decreases cell viability.**

(**A**) Representative crystal violet staining after 72 h KLF5 knockdown in AsPC1, HPAFII, BxPC3, L3.6pl and immortalized normal human pancreatic ductal epithelial cell line (HPDEC). (**B**) Quantification of cell confluency of the conditions described in (A). Unpaired Student's t-test, p values shown on graph. n = 3 biological replicates. (**C**) Representative Western blot analysis of KLF5 after 72 h KLF5 knockdown. HSC70 was used as a loading control.

fig. S10

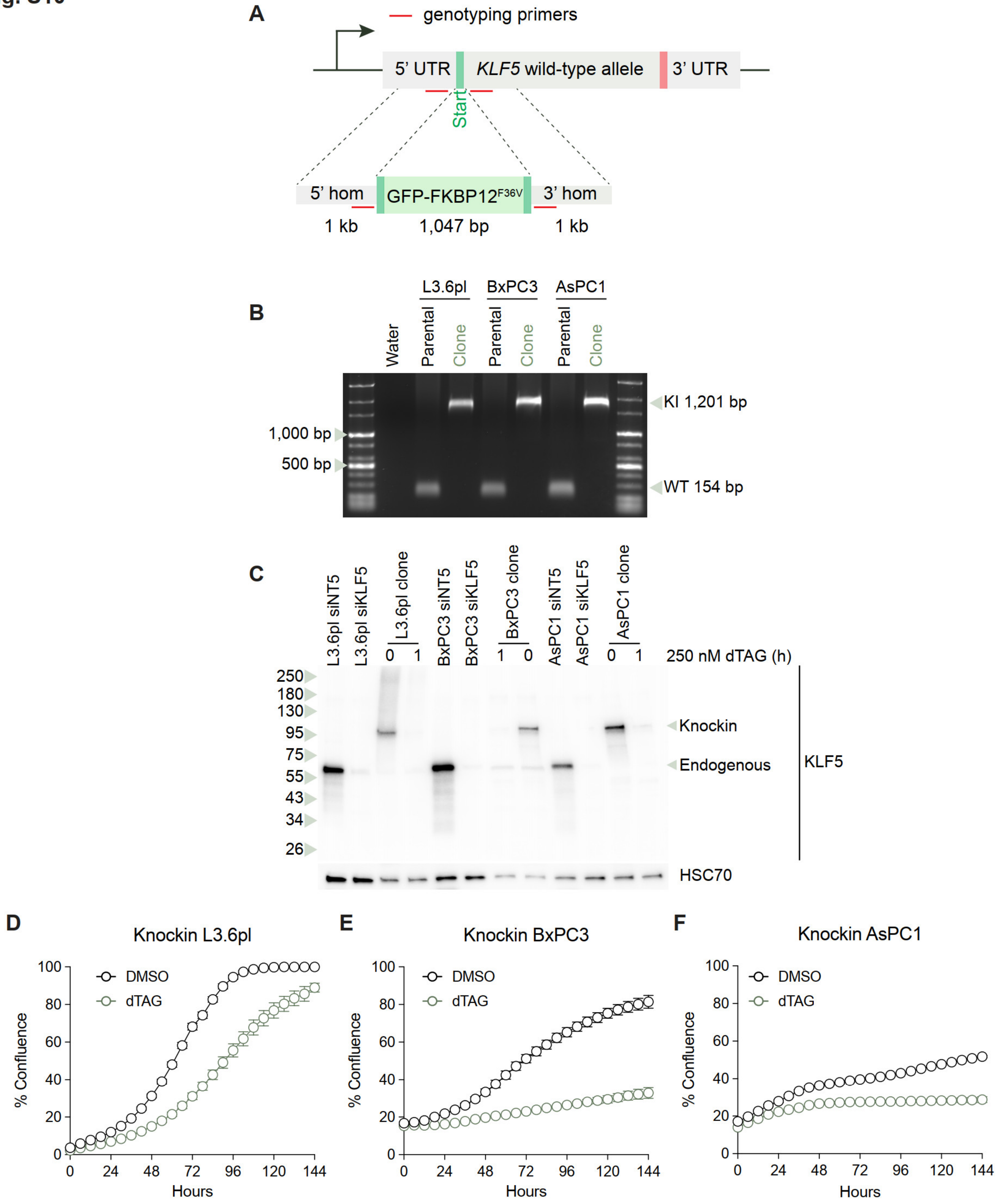


**fig. S10. Generation of KLF5 degron cell lines.**

(**A**) Schematic depicting genotyping approach for the endogenous knock-in of GFP-FKBP12$^{F36V}$ into the N-terminus of the *KLF5* locus. (**B**) Representative genotyping PCR for N-terminus knockin of *KLF5*. WT band (154 bp) and knockin band (1201 bp) show homozygous knockin in L3.6pl, BxPC3 and AsPC1 cell lines. (**C**) Representative Western blot of KLF5 in clonal knockin cell lines. Observed endogenous KLF5 molecular weight (~55 kDa), EGFP (~26 kDa) and FKBP12$^{F36V}$ (~12 kDa). HSC70 was used as a loading control. (**D to F**) Knockin L3.6pl (E), BxPC3 (F) and AsPC1 (G) cells were plated overnight and treated with 250 nM dTAG and monitored via live cell imaging for proliferation.

fig. S11

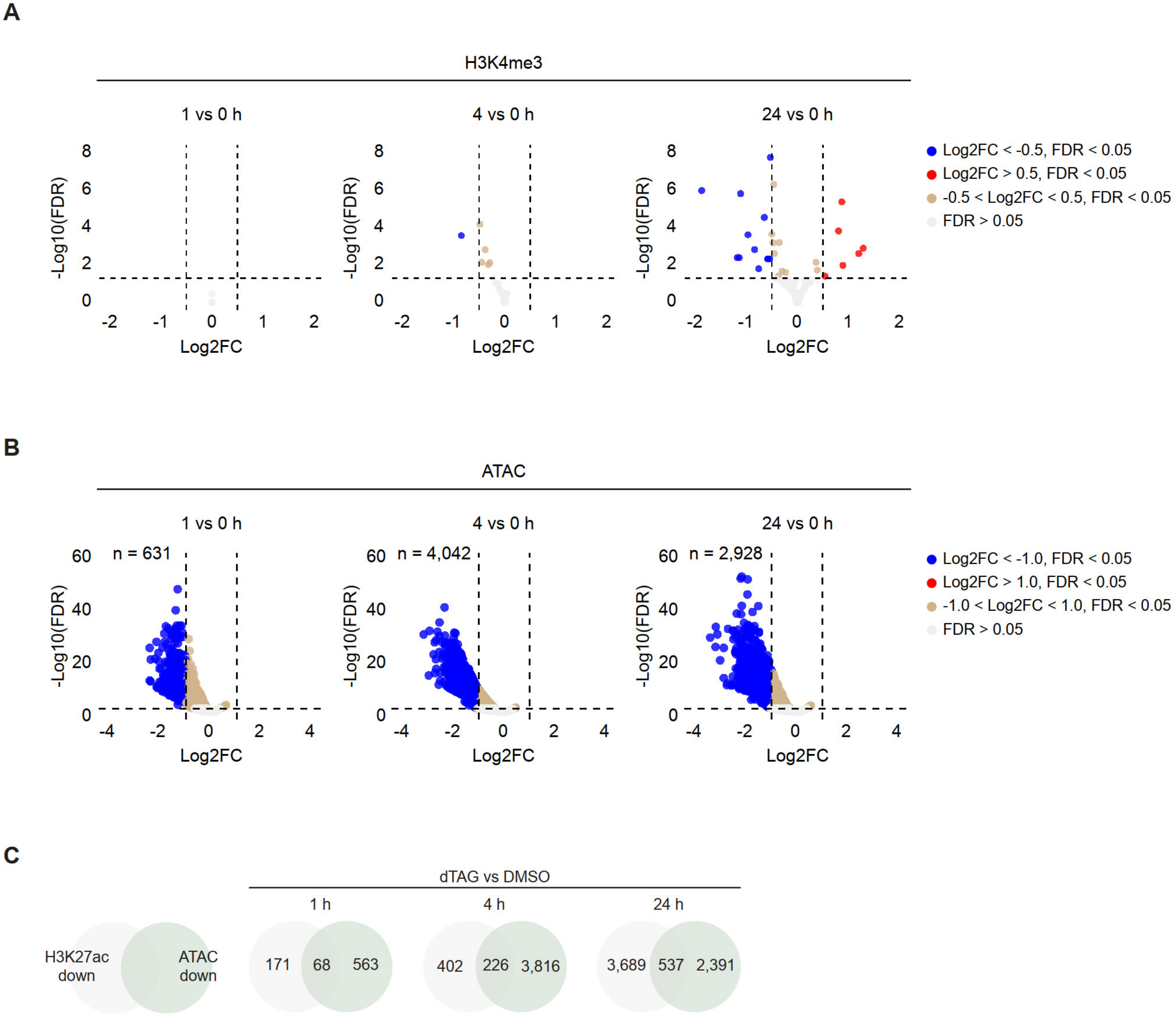


**fig. S11. Effect of KLF5 degradation on H3K4me3 and ATAC levels.**

(**A and B**) Differential binding analysis of genome-wide H3K4me3 ChIP-seq (A) or chromatin accessibility (B) in knockin L3.6pl cells upon 1, 4 and 24 h dTAG treatment. n = 3 biological replicates. (**C**) Overlap of differentially bound H3K27ac and open regions in knockin L3.6pl cells upon 1, 4 and 24 h dTAG treatment.

## fig. S12

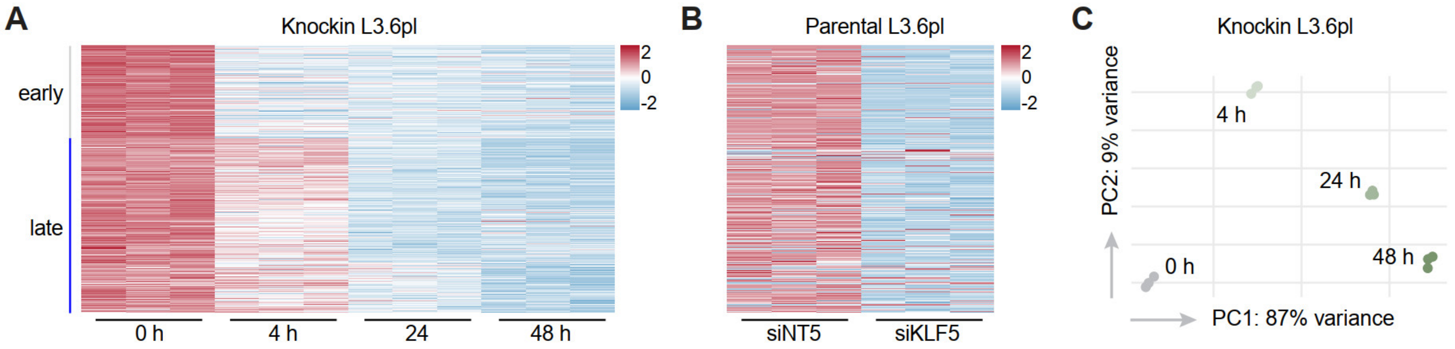


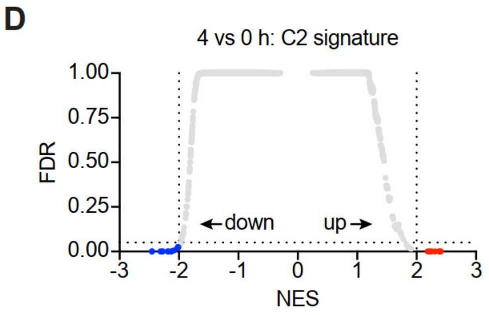


| Common regulated gene sets | | | |
|---|---|---|---|
| Anderson cholangiocarcinoma class 2 | -2.45 | Reactome of the cornified envelope | -2.12 |
| Reactome keratinization | -2.27 | Cromer metastasis down | -2.27 |

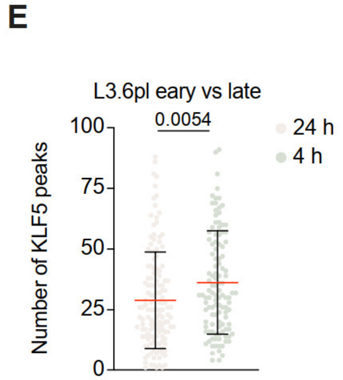


**fig. S12. Early KLF5-dependent genes connect with multiple KLF5 peaks.**

(**A**) Heatmap shows the raw z-score values generated on vst transformed normalized counts from RNA-seq after 4, 24 and 48 h dTAG treatment in knockin L3.6pl cells. n = 3 biological replicates. (**B**) Heatmap of raw z-score values generated on vst transformed normalized counts from RNA-seq upon 24 h KLF5 knockdown in parental L3.6pl cells ranked on genes in (A). n = 3 biological replicates. (**C**) Principal component analysis (PCA) in knockin L3.6pl cells after 4, 24, and 48 h dTAG treatment as described in (A). (**D**) Gene Set Enrichment Analysis after 4 h dTAG treatment in knockin L3.6pl cells. Normalized enrichment score is on x-axis and false discovery rate on y-axis. Significantly downregulated genes overlap with pathways regulated upon KLF5 knockdown in parental L3.6pl cells. (**E**) Cumulative percentage plots of the number of KLF5 peaks that interact with H3K4me3 marked early (4 h) or late (24 h) KLF5-dependent genes (Log2FC < -0.5, FDR < 0.05). 139 (4 h) and 179 (24 h) genes were included in the analysis. Unpaired Student's t test, **** $p < 0.0001$.

fig. S13

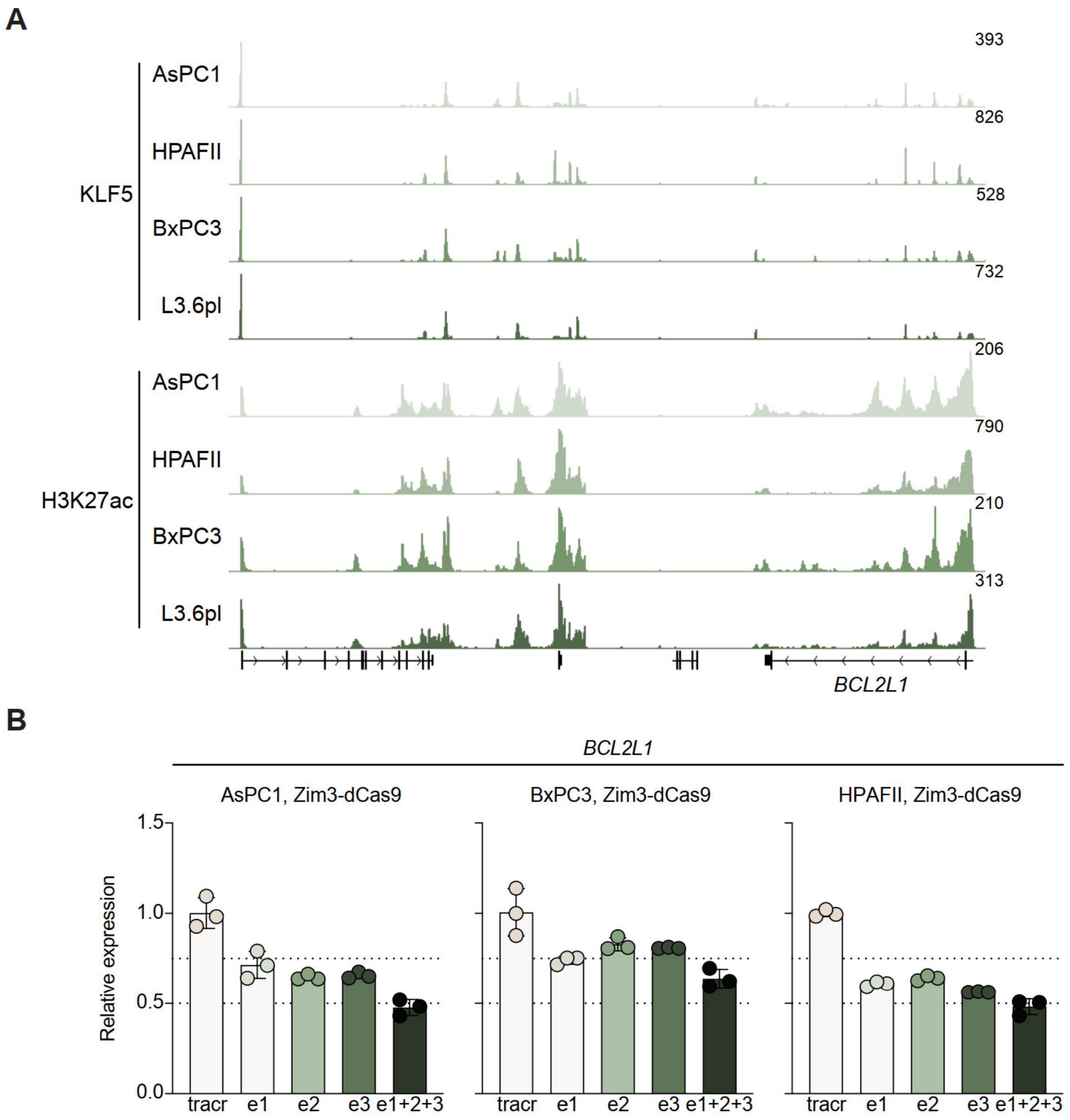


**fig. S13. Downstream enhancers of *BCL2L1* are dependent on KLF5 and regulate gene expression.**

(**A**) Integrated genome viewer (IGV) track of the *BCL2L1* locus showing similar KLF5 and H3K27ac occupancy across multiple KLF5-dependent cell lines AsPC1, HPAFII, BxPC3 and L3.6pl cells. (**B**) qPCR of *BCL2L1* upon 48 h Zim3-dCas9 simultaneously targeting all three marked enhancers in AsPC1, BxPC3 and HPAFII cells. *ACTB* was used to normalize gene expression. n = 3 biological replicates.

## fig. S14

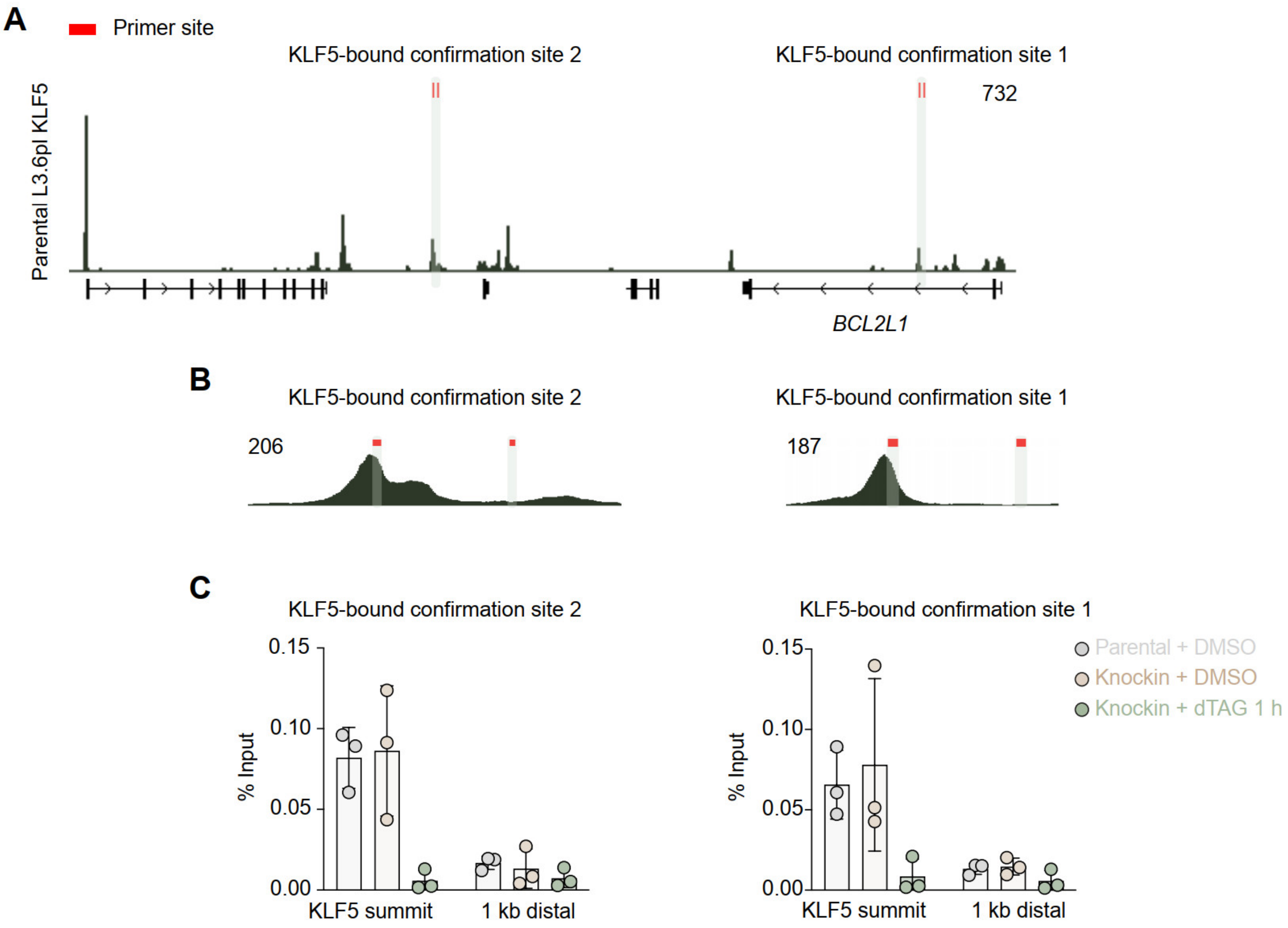


**fig. S14. Fusion of GFP-dTAG to KLF5 does not alter KLF5 binding.**

(**A**) Zoomed out integrated genome viewer (IGV) track showing ChIP-seq of KLF5 in parental L3.6pl cells at the *BCL2L1* locus. Red bar and green shading represent primer binding sites. (**B**) Zoomed in IGV track showing primers bind either the KLF5 summit or 1 kb downstream of the KLF5 summit at two loci. (**C**) ChIP-qPCR of KLF5 in parental or knockin L3.6pl cells with DMSO of knockin L3.6pl cells after 1 h dTAG treatment using an antibody targeting the endogenous KLF5.

fig. S15

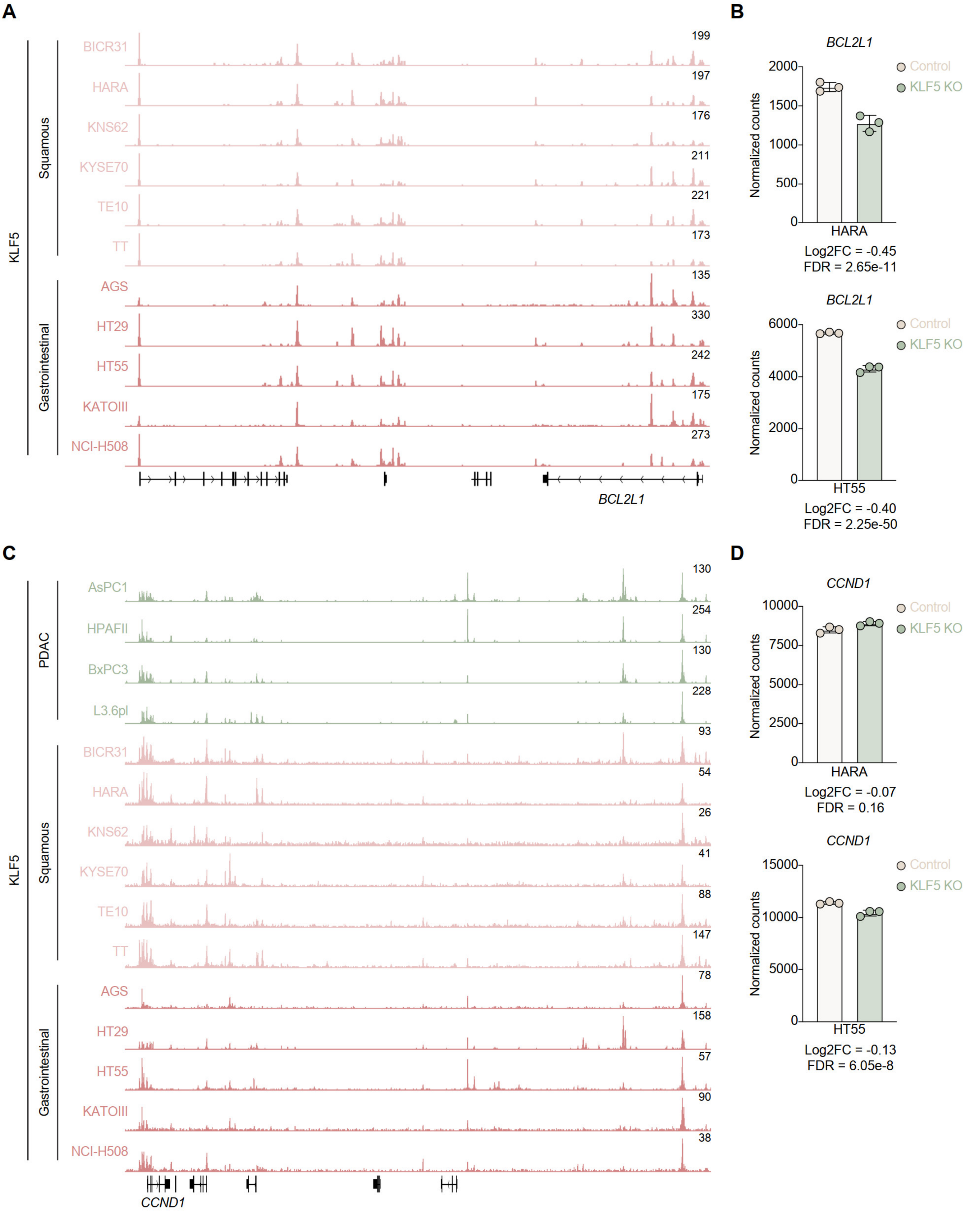

A
KLF5
Squamous
Gastrointestinal
BICR31
199
HARA
197
KNS62
176
KYSE70
211
TE10
221
TT
173
AGS
135
HT29
330
HT55
242
KATOIII
175
NCI-H508
273
BCL2L1
B
BCL2L1
Normalized counts
2000
1500
1000
500
0
Control
KLF5 KO
HARA
Log2FC = -0.45
FDR = 2.65e-11
BCL2L1
6000
4000
2000
0
HT55
Log2FC = -0.40
FDR = 2.25e-50
C
KLF5
PDAC
Squamous
Gastrointestinal
AsPC1
130
HPAFII
254
BxPC3
130
L3.6pl
228
BICR31
93
HARA
54
KNS62
26
KYSE70
41
TE10
88
TT
147
AGS
78
HT29
158
HT55
57
KATOIII
90
NCI-H508
38
CCND1
D
CCND1
10000
7500
5000
2500
0
HARA
Log2FC = -0.07
FDR = 0.16
CCND1
15000
10000
5000
0
HT55
Log2FC = -0.13
FDR = 6.05e-8

## fig. S15

**fig. S15. *BCL2L1* and *CCND1* are regulated via KLF5 in additional non-pancreatic cancer KLF5-dependent cell systems.**

(**A and C**) Integrated genome viewer (IGV) tracks of publicly available KLF5 ChIP-seq data (PMID: 33115806) in squamous cancer cell lines (light pink) and gastrointestinal cancer cell lines (dark pink) showing colocalization with in-house KLF5 ChIP-seq in pancreatic cancer cell lines (green) at the KLF5-dependent enhancers for *BCL2L1* (A) and *CCND1* (C). (**B and D**) Analysis of publicly available RNA-seq data showing normalized counts of *BCL2L1* (B) and *CCND1* (D) after KLF5 knockout in HARA (squamous) and HT55 (gastro-intestinal) cell lines. Log2FC and FDR are displayed below each graph.

fig. S16

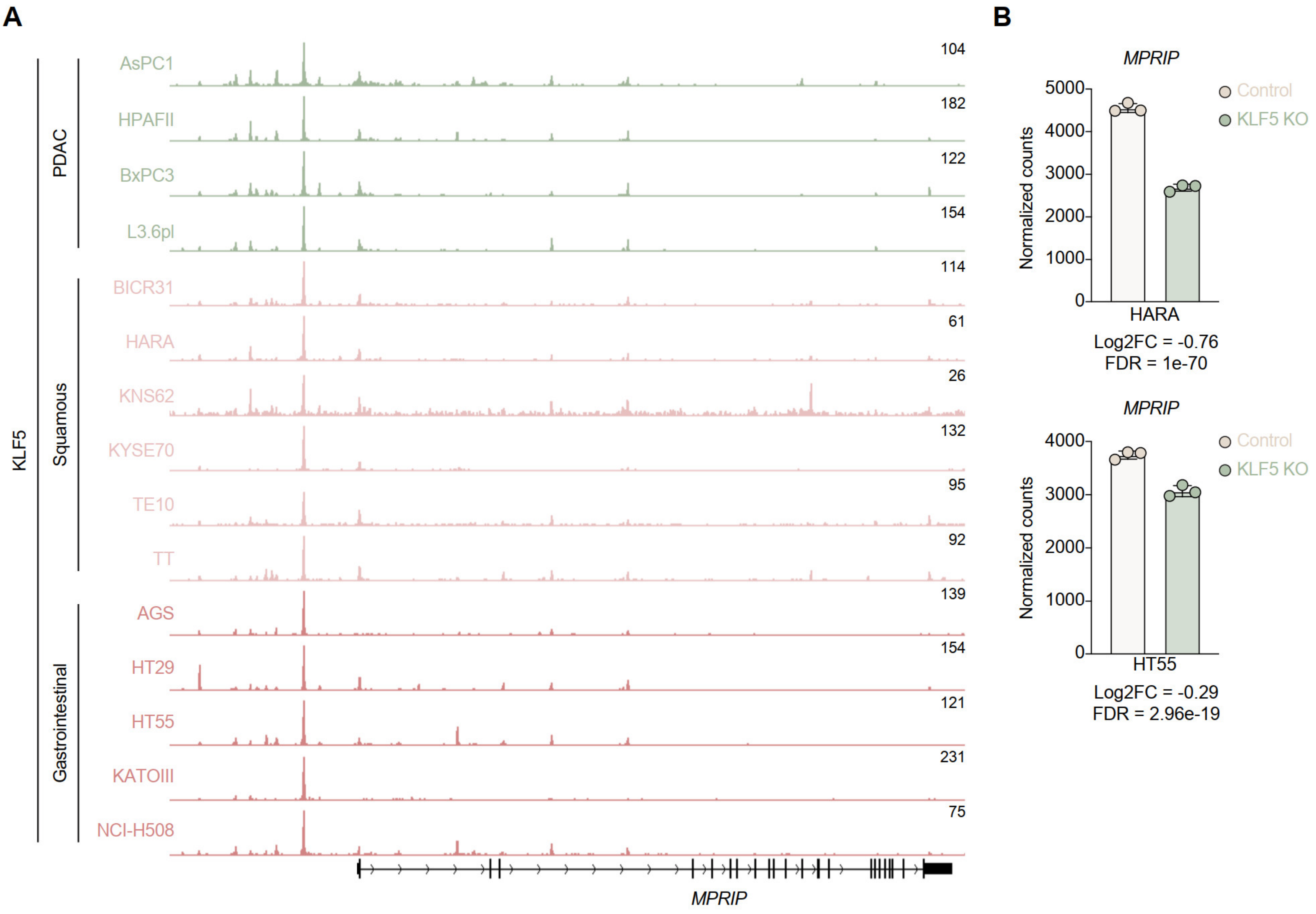


**fig. S16. *MPRIP* is regulated via KLF5 in additional non-pancreatic cancer KLF5-dependent cell systems.**

(**A**) Integrated genome viewer (IGV) tracks of publicly available KLF5 ChIP-seq data (PMID: 33115806) in squamous cancer cell lines (light pink) and gastrointestinal cancer cell lines (dark pink) showing colocalization with in-house KLF5 ChIP-seq in pancreatic cancer cell lines (green) at the KLF5-dependent enhancers for *MPRIP*. (**B**) Analysis of publicly available RNA-seq data showing normalized counts of *MPRIP* after KLF5 knockout in HARA (squamous) and HT55 (gastrointestinal) cell lines. Log2FC and FDR are displayed below each graph.

fig. S17

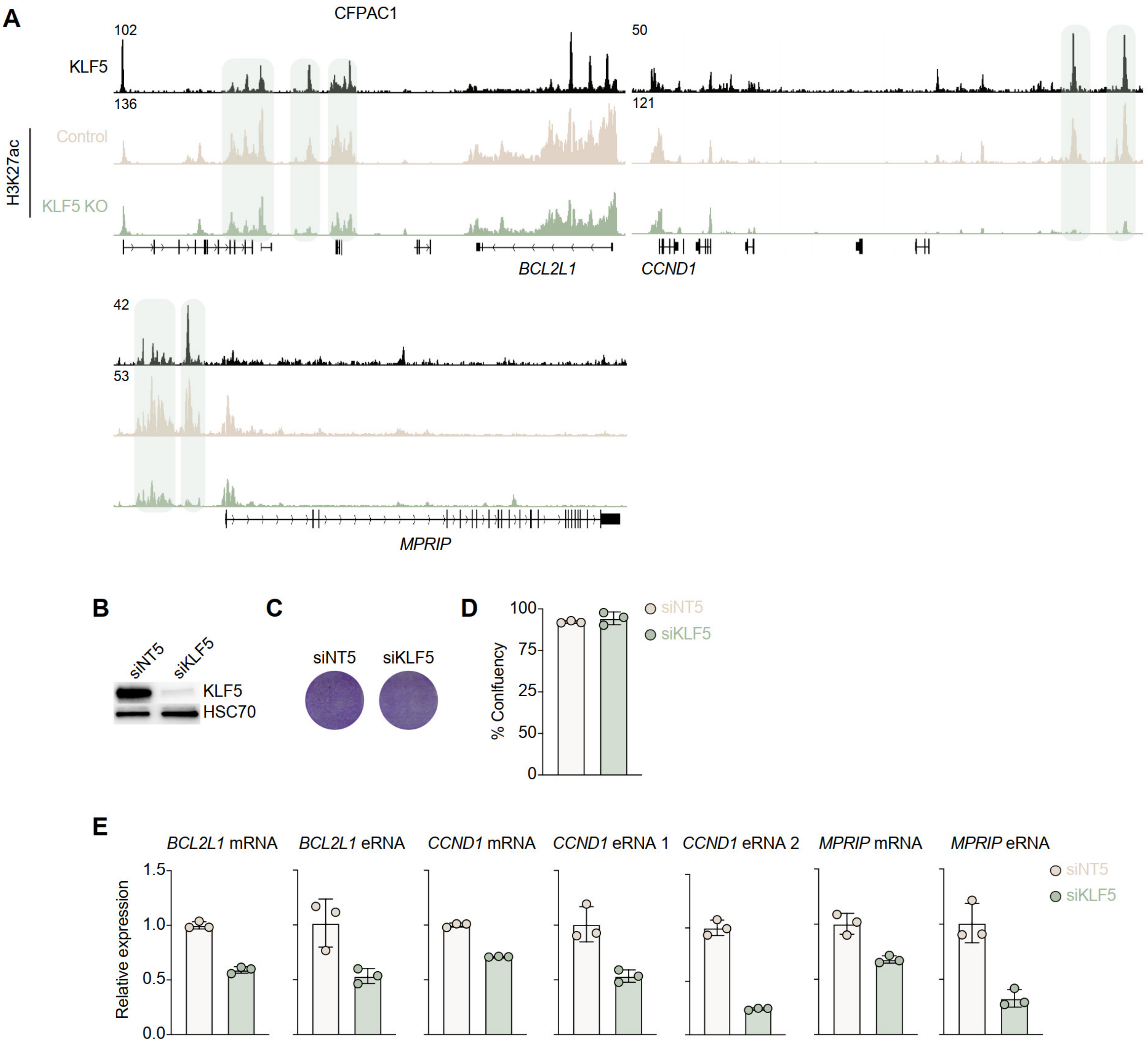


**fig. S17. KLF5 regulates similar enhancers and genes in KLF5-independent cell lines.**

(**A**) Integrated genome viewer (IGV) tracks of publicly available KLF5 and H3K27ac ChIP-seq data (PMID: 26769127) in KLF5-independent CFPAC1 cells showing KLF5 knockout decreases H3K27ac levels at the enhancers for *BCL2L1* (top left), *CCND1* (top right) and *MPRIP* (bottom left) and is bound by KLF5. (**B**) Representative Western blot in CFPAC1 cells after 24 h KLF5 knockdown. HSC70 was used as a loading control. (**C**) Representative crystal violet staining in CFPAC1 cells after KLF5 knockdown for 72 h. (**D**) Quantification of cell confluency of the conditions described in (C). n = 3 biological replicates. (**E**) qPCR of *BCL2L1*, *CCND1* and *MPRIP* mRNA and eRNA after 24 h knockdown in CFPAC1 cells. *ACTB* was used to normalize gene expression. n = 3 biological replicates.

fig. S18

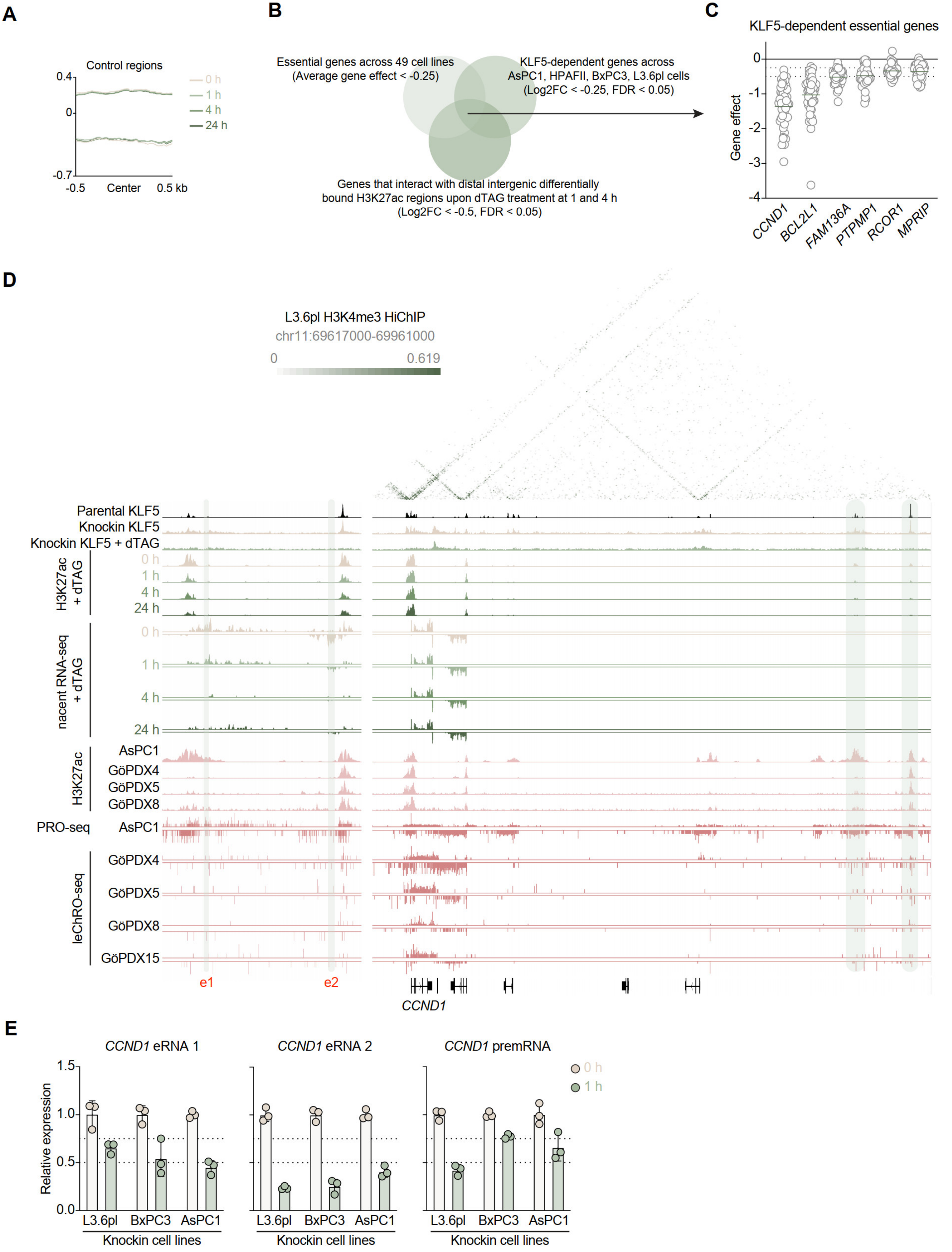

A
Control regions
0.4
0
-0.7
-0.5
Center
0.5 kb
0 h
1 h
4 h
24 h
B
Essential genes across 49 cell lines
(Average gene effect < -0.25)
KLF5-dependent genes across
AsPC1, HPAFII, BxPC3, L3.6pl cells
(Log2FC < -0.25, FDR < 0.05)
Genes that interact with distal intergenic differentially
bound H3K27ac regions upon dTAG treatment at 1 and 4 h
(Log2FC < -0.5, FDR < 0.05)
C
KLF5-dependent essential genes
Gene effect
0
-1
-2
-3
-4
CCND1
BCL2L1
FAM136A
PTPMP1
RCOR1
MPRIP
D
L3.6pl H3K4me3 HiChIP
chr11:69617000-69961000
0
0.619
Parental KLF5
Knockin KLF5
Knockin KLF5 + dTAG
H3K27ac
+ dTAG
0 h
1 h
4 h
24 h
nacent RNA-seq
+ dTAG
0 h
1 h
4 h
24 h
H3K27ac
AsPC1
GöPDX4
GöPDX5
GöPDX8
PRO-seq
AsPC1
leChRO-seq
GöPDX4
GöPDX5
GöPDX8
GöPDX15
e1
e2
CCND1
E
CCND1 eRNA 1
CCND1 eRNA 2
CCND1 premRNA
0 h
1 h
Relative expression
1.5
1.0
0.5
0.0
L3.6pl
BxPC3
AsPC1
Knockin cell lines

## fig. S18

**fig. S18. KLF5 regulates *CCND1* gene and eRNA transcription across pancreatic cancer cell lines.**

(**A**) Aggregate plot of nascent RNA-seq in knockin L3.6pl cells after 0, 1, 4 and 24 h dTAG treatment on control regions identified. Regions are centered on the KLF5 summit. cpm normalized, 516 regions were plotted, n = 3 biological replicates. (**B**) Schematic showing the strategy for enhancer RNAs that stem from enhancers that are KLF5-dependent and interact with essential genes that are regulated via KLF5 across multiple pancreatic cancer cell lines. (**C**) Gene effect of genes identified in (A) across 47 pancreatic cancer cell lines from the CRISPR-dependency data from DepMap data. (**D**) Integrated genome viewer (IGV) showing KLF5 and H3K27ac ChIP-seq and nascent RNA profiling in knockin L3.6pl cells after dTAG treatment and H3K27ac ChIP-seq and run-on sequencing in AsPC1 cells and patient-derived xenografts (GöPDX4, GöPDX15, GöPDX5, GöPDX8) at the *MPRIP* locus. Green shading highlights the enhancers which are transcribed and KLF5-dependent. ChIP-seq reads normalized to reads per genome coverage (RPGC) and nascent RNA normalized to counts per million (cpm). (**E**) qPCR of *MPRIP* eRNA (left) and premRNA (right) in knockin L3.6pl, BxPC3 and AsPC1 cells after 1 h dTAG treatment. *ACTB* was used to normalize gene expression. n = 3 biological replicates.

**fig. S19**

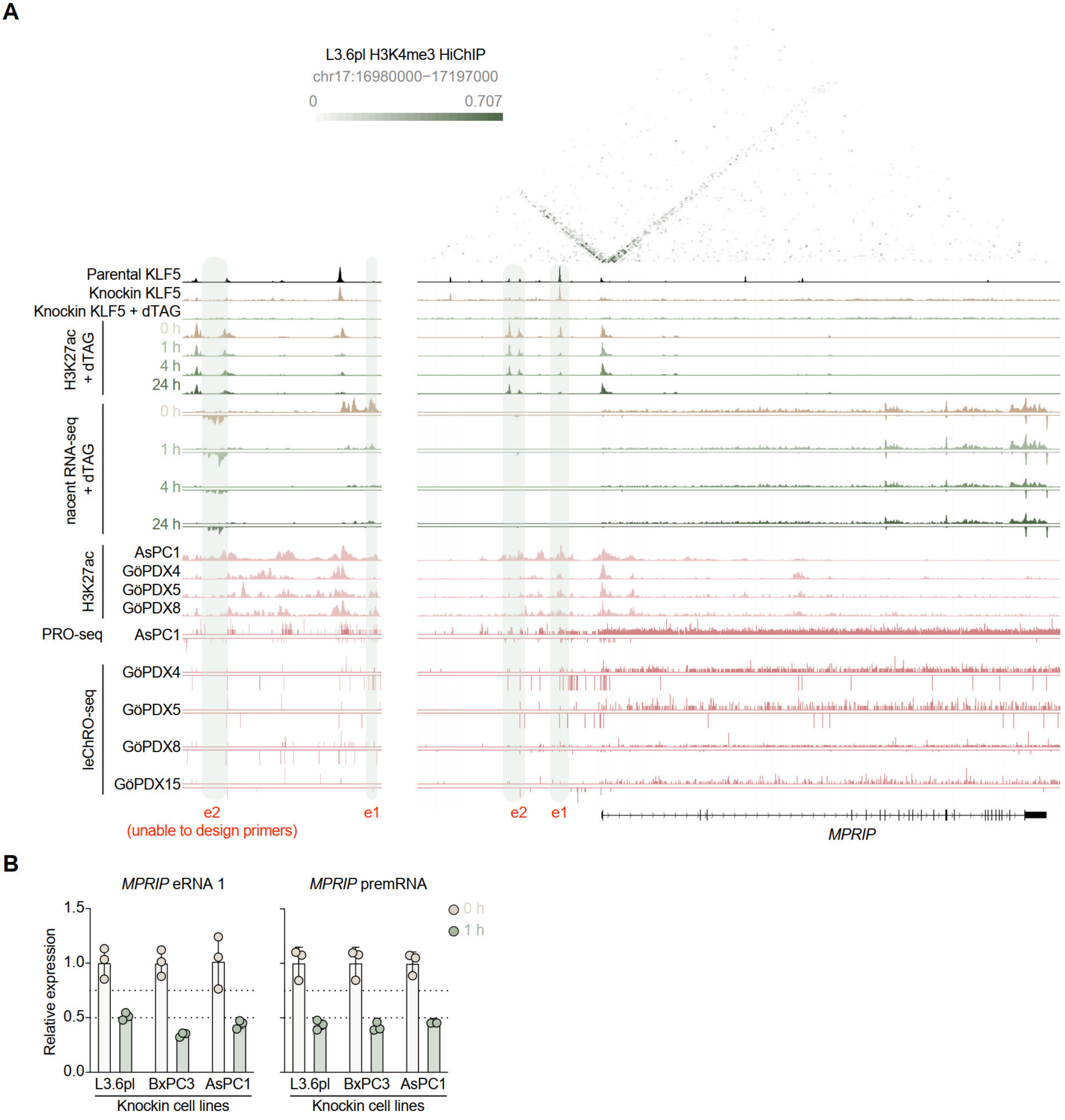


**fig. S19. KLF5 regulates *MPRIP* gene and eRNA transcription across pancreatic cancer cell lines.**

(**A**) Integrated genome viewer (IGV) showing KLF5 and H3K27ac ChIP-seq and nascent RNA profiling in knockin L3.6pl cells after dTAG treatment and H3K27ac ChIP-seq and run-on sequencing in AsPC1 cells and patient-derived xenografts (GöPDX4, GöPDX15, GöPDX5, GöPDX8) at the *MPRIP* locus. Green shading highlights the enhancers which are transcribed and KLF5-dependent. ChIP-seq reads normalized to reads per genome coverage (RPGC) and nascent RNA normalized to counts per million (cpm). (**B**) qPCR of *MPRIP* eRNA (left) and premRNA (right) in knockin L3.6pl, BxPC3 and AsPC1 cells after 1 h dTAG treatment. *ACTB* was used to normalize gene expression. n = 3 biological replicates.

fig. S20

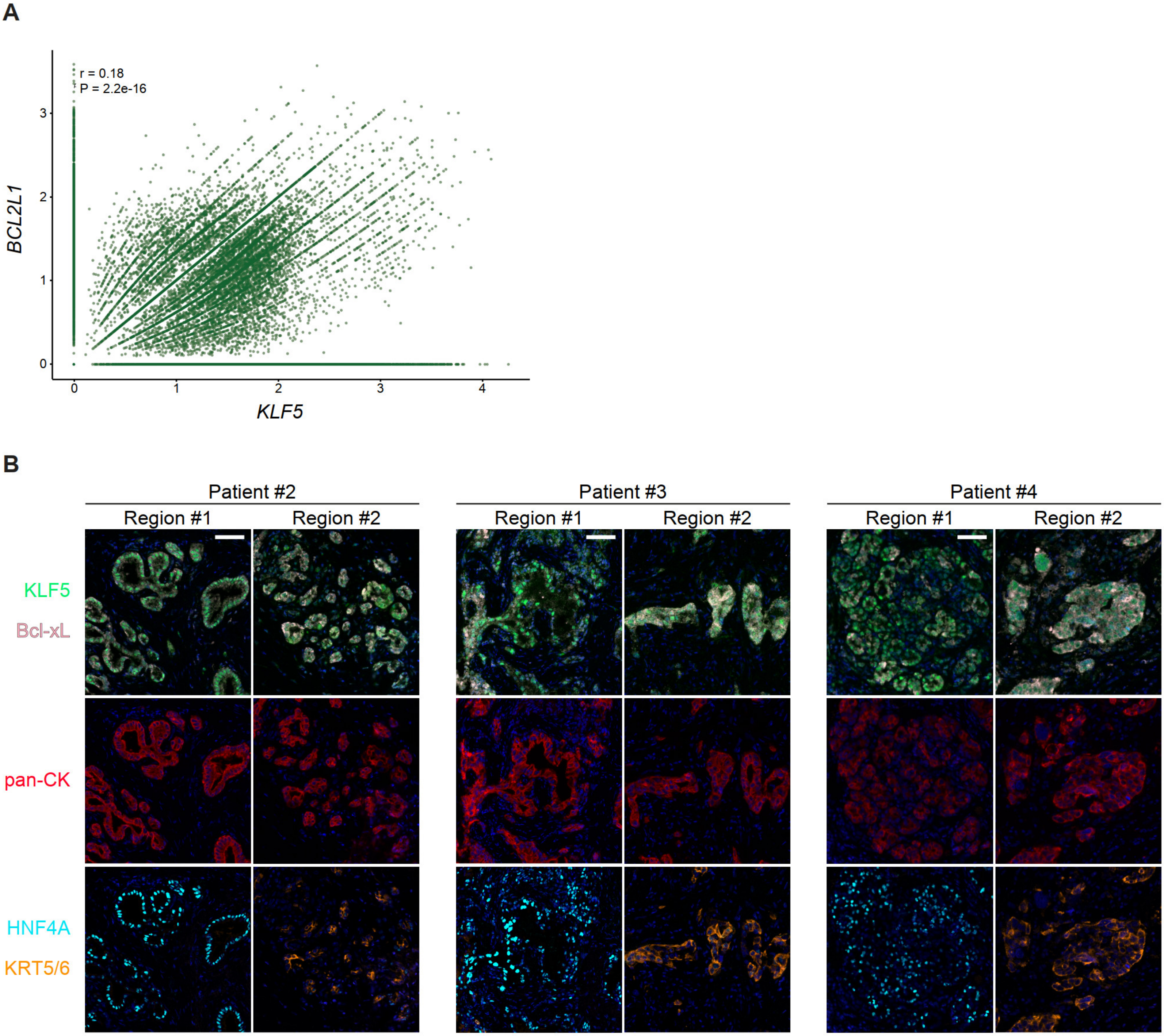


**fig. S20. KLF5 and Bcl-xL are co-expressed in patients.**

(**A**) Pearson correlation of *KLF5* and *BCL2L1* gene expression in patient single-cell RNA-seq data. (**B**) Representative multiplex immunofluorescence images validating KLF5 and Bcl-xL co-expression in patient samples independent of subtype-identity. Scale bar represents 50 µm.

**fig. S21**

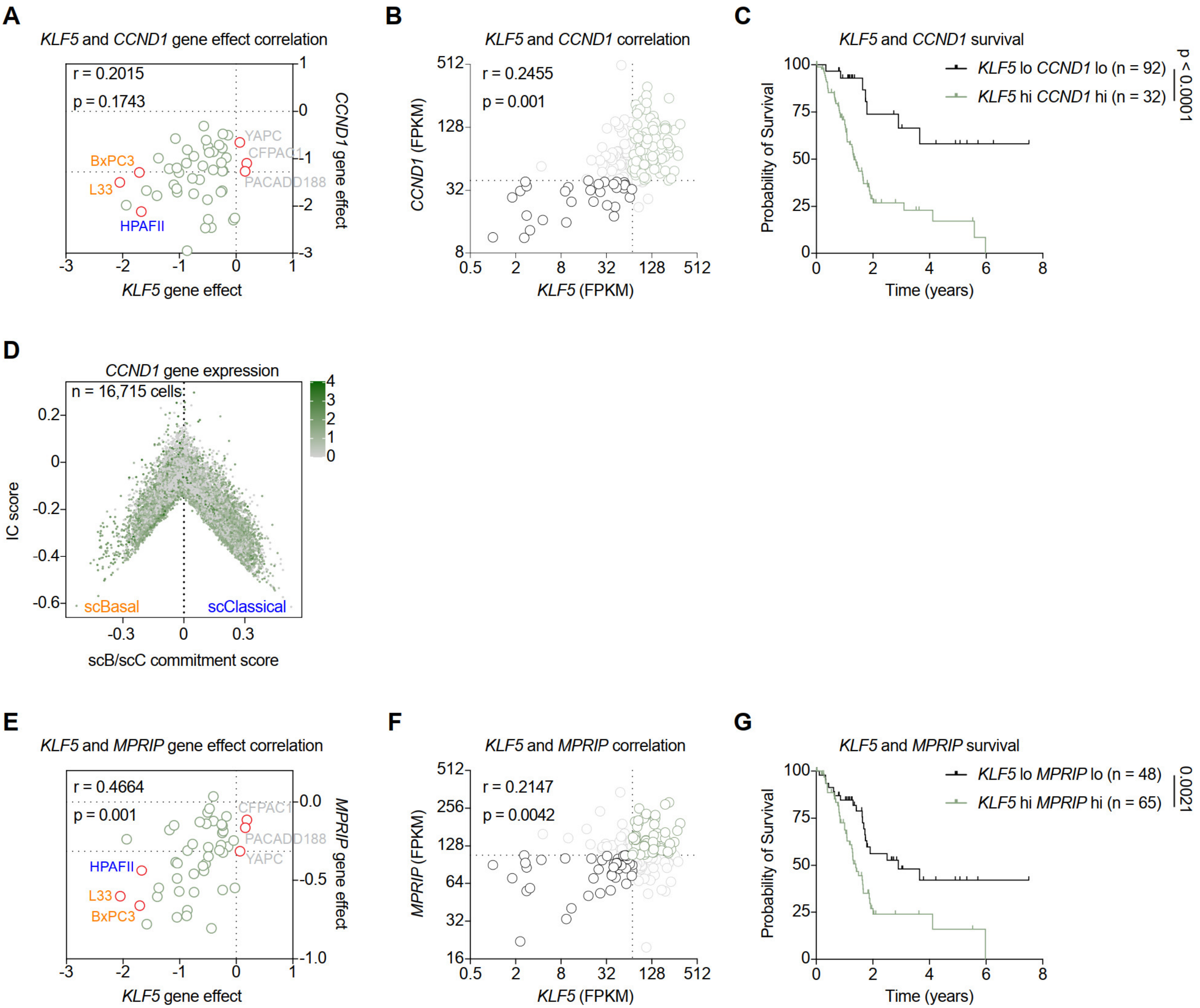


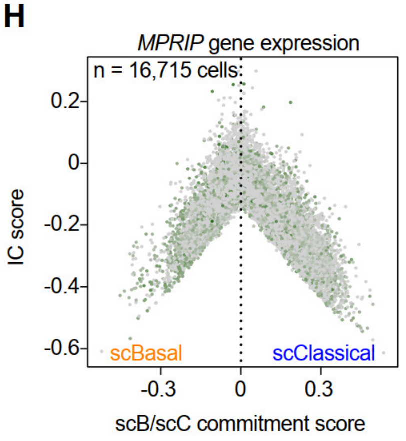


**fig. S21. Gene expression of KLF5-target genes *CCND1* and *MPRIP* are correlated with *KLF5* in patients.**

(**A and E**) Correlation of *KLF5* and *CCND1* (A) and *MPRIP* (E) gene effect from the CRISPR-dependency data from DepMap data. (**B and F**) Correlation of *KLF5* and *CCND1* (B) and *MPRIP* (F) gene expression in pancreatic cancer patients from The Cancer Genome Atlas (TCGA) database. FPKM cut off values 70.84, 39.72 and 108.05 were used for *KLF5*, *CCND1* and *MPRIP*, respectively. (**C and G**) Kaplan-Meier plot of *KLF5*-hi, *CCND1*/*MPRIP*-hi vs *KLF5*-lo, *CCND1*/*MPRIP*-lo patients using the cut off values from (B and F). (**D and H**) Cell state diagram on the tumorigenic cells showing *KLF5* and *CCND1* (D) and *MPRIP* (H) expression in scRNA-seq. scBasal-scClassical commitment score (x-axis) and intermediate co-expressor score (y-axis).

**fig. S22**

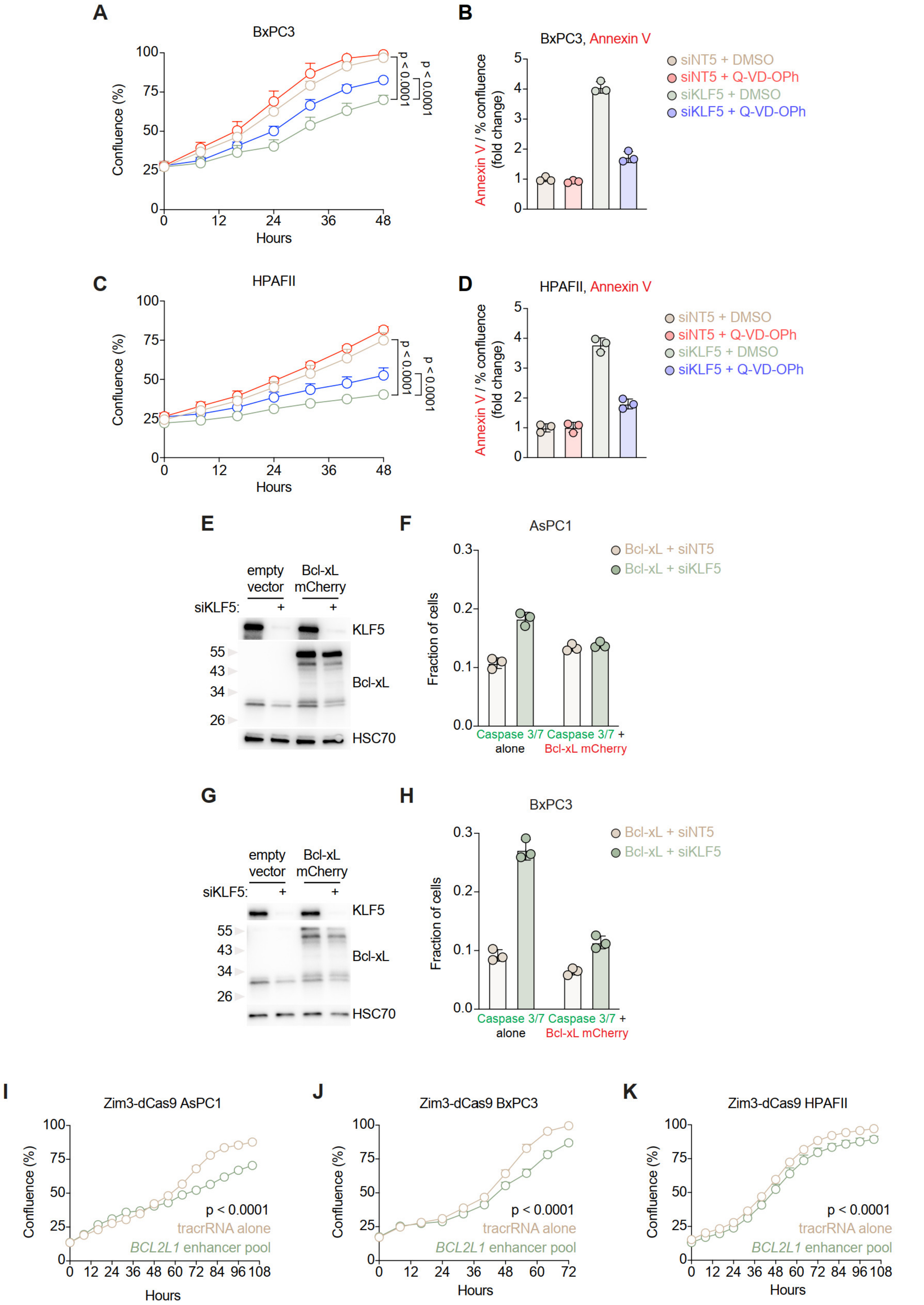
A
BxPC3
Confluence (%)
Hours
p < 0.0001
p < 0.0001
B
BxPC3, Annexin V
Annexin V / % confluence (fold change)
siNT5 + DMSO
siNT5 + Q-VD-OPh
siKLF5 + DMSO
siKLF5 + Q-VD-OPh
C
HPAFII
Confluence (%)
Hours
p < 0.0001
p < 0.0001
D
HPAFII, Annexin V
Annexin V / % confluence (fold change)
siNT5 + DMSO
siNT5 + Q-VD-OPh
siKLF5 + DMSO
siKLF5 + Q-VD-OPh
E
empty vector
Bcl-xL mCherry
siKLF5:
KLF5
Bcl-xL
HSC70
F
AsPC1
Fraction of cells
Bcl-xL + siNT5
Bcl-xL + siKLF5
Caspase 3/7 alone
Caspase 3/7 + Bcl-xL mCherry
G
empty vector
Bcl-xL mCherry
siKLF5:
KLF5
Bcl-xL
HSC70
H
BxPC3
Fraction of cells
Bcl-xL + siNT5
Bcl-xL + siKLF5
Caspase 3/7 alone
Caspase 3/7 + Bcl-xL mCherry
I
Zim3-dCas9 AsPC1
Confluence (%)
Hours
p < 0.0001
tracrRNA alone
BCL2L1 enhancer pool
J
Zim3-dCas9 BxPC3
Confluence (%)
Hours
p < 0.0001
tracrRNA alone
BCL2L1 enhancer pool
K
Zim3-dCas9 HPAFII
Confluence (%)
Hours
p < 0.0001
tracrRNA alone
BCL2L1 enhancer pool

## fig. S22

**fig. S22. KLF5 regulates cell viability partially through apoptosis.**

(**A to D**) BxPC3 and HPAFII cells were treated with KLF5 or non-targeting siRNA mix, incubated for 4 h and media was changed with DMSO or 10 μM Q-VD-OPh with live cell annexin V reagent (1:1,500) and monitored via live cell imaging for proliferation and red calibrated units (RCU). n = 3 biological replicates. (A and C) Quantification of confluency over time. Unpaired Student's t-test on the area under the curve (AUC), p values shown on graph. (B and D) Fold change (siKLF5 vs siNT5) of RCU per % confluency. (**E to H**) AsPC1 and BxPC3 cells were transfected with either empty vector backbone (pcDNA3.1) or containing Bcl-xL-mCherry for 24 h and subsequently transfected with KLF5 or non-targeting siRNA mix, incubated for 4 h and media was changed with live cell caspase 3/7 reagent (1:1,000) and monitored via live cell imaging for proliferation and GCU and RCU. n = 3 biological replicates. (H and J) Representative Western blot analysis of KLF5 and Bcl-xL confirming knockdown and overexpression, respectively. HSC70 was used as a loading control. (I and K) Fraction of cells with only caspase3/7 reagent (GCU) or caspase 3/7 reagent and mCherry (GCU + RCU). (**I to K**) Lentiviral Zim3-dCas9 AsPC1 (A), BxPC3 (F) and HPAFII (G) cells were treated with a pool targeting downstream enhancers of *BCL2L1* or tracrRNA alone and monitored via live cell imaging for proliferation. n = 3 biological replicates. Quantification of confluency over time. Unpaired Student's t-test on the AUC, p values shown on graph.

## fig. S23

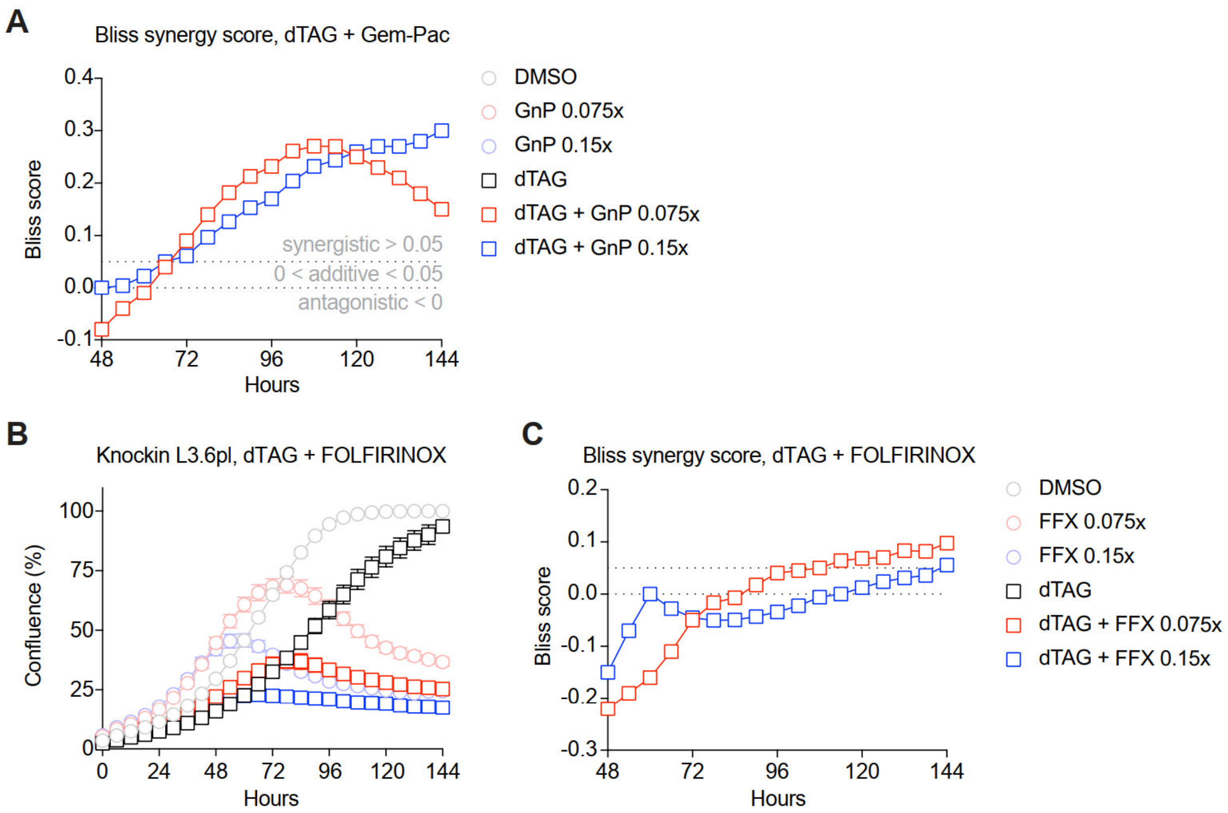


**fig. S23. KLF5 degradation synergizes with gemcitabine-paclitaxel.**

(**A**) Quantification of Bliss synergy score from (Fig 5L). (**B and C**) Knockin L3.6pl cells were plated overnight, treated with indicated reagents and monitored for confluency. n = 6 biological replicates. (B) Quantification of confluency over time. (C) Quantification of Bliss synergy score.

## fig. S24

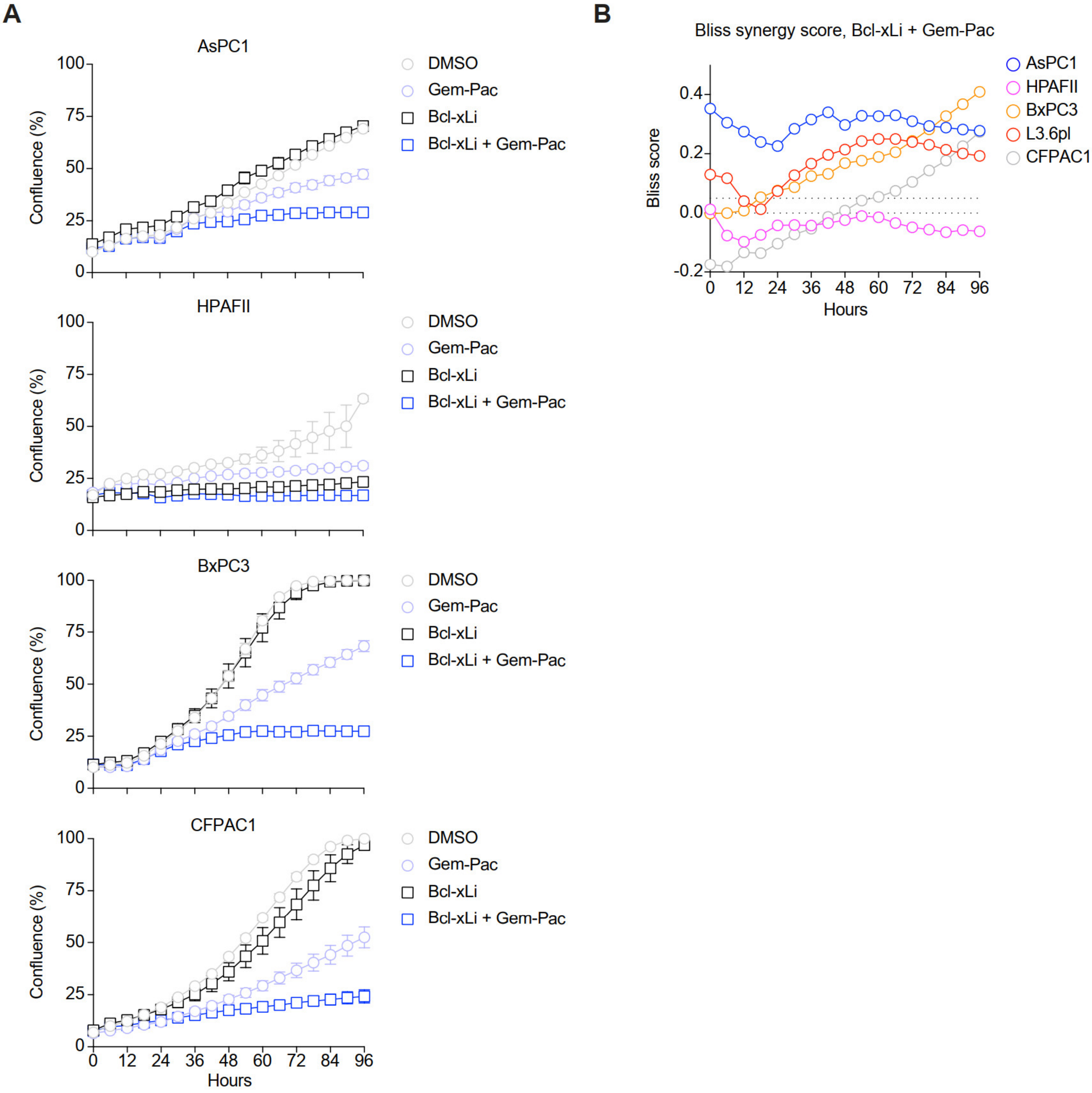


**fig. S24. Bcl-xL inhibition synergizes with gemcitabine-paclitaxel.**

(**A**) Cells were plated overnight, co-treated with gemcitabine/paclitaxel and A-1155643 and monitored for confluency. n = 3 biological replicates. Cell lines were treated with gemcitabine/paclitaxel and A-1155643 as follows: AsPC1 (0.15x, 1 μM), HPAFII (0.075x, 1 nM), BxPC3 (0.075x, 10 μM), CFPAC1 (0.3x, 10 nM). (**B**) Quantification of Bliss synergy score from (A).

## fig. S25

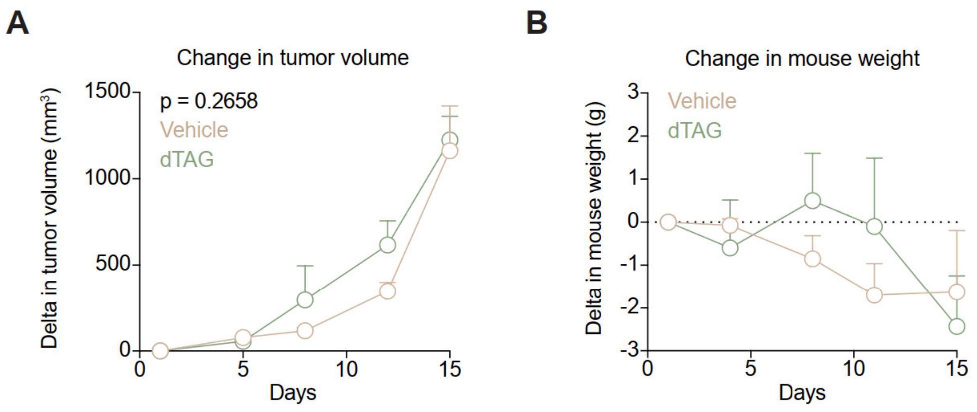


**fig. S25. KLF5 regulates cell viability *in vivo*.**

(A) Change in tumor volume, n = 3 mice. Unpaired Student's t-test on the area under the curve (AUC), p values shown on graph. (B) Change in mouse weight.

| Primer | | | |
|---|---|---|---|
| **Application** | **Target** | **Forward** | **Reverse** |
| mRNA expression | ACTB | CACCATTGGCAATGAGCGGTTC | AGGTCTTTGCGGATGTCCACGT |
| | BCL2L1 | TGCAGGTATTGGTGAGTCGG | CACAAAAGTATCCCAGCCGC |
| | CCND1 | ATCAAGTGTGACCCGGACTG | CTTGGGGTCCATGTTCTGCT |
| | KLF5 | CGATTTGGAGAAACGACGCA | TTGTATGGCTTTTCACCAGTGTG |
| | MPRIP | CCACCATCTCAGCCATCGAA | CAACATCCGAGTTGACGCTG |
| ChIP qPCR | KLF5-bound confirmation site 1 positive | CCAATCTTCCGTTTACACAGGC | TTCCTGTAGGCTAGCTTTGAACT |
| | KLF5-bound confirmation site 1 negative | CTCACTGGGGCTTGGGATAC | GGGGGAACTTTCTGGGTCTG |
| | KLF5-bound confirmation site 2 positive | CATTCAACCTCAGCTGGTTCC | CTCCCATATAACTGGCTTCCCTC |
| | KLF5-bound confirmation site 2 negative | TGCATGTGGTTCTGTTAGGAGT | GAGGAAATGTCTCAAAACACCCC |
| premRNA | BCL2L1 | CACAAGGGGCTTGGTTCTTAC | GATGGCCACTTACCTGAATGAC |
| | CCND1 | ACTTCAAATGTGTGCAGAAGGAG | GGGGTGAGTAGCAAAGAAACG |
| | MPRIP | TCAACGACGAGGACCTGAC | CTGCTCCATTTTTATTTTCCCTGC |
| eRNA | BCL2L1 | AGCCTACAAGGCCAAGACTC | GTTGACGCACTGTTCTCGTC |
| | CCND1 enhancer 1 | CGTTTGACACGGCCTTTCC | CTGACCCTACAGCCCAACTC |
| | CCND1 enhancer 2 | GATGCTCCAAATCAGGGAAACC | GTCCACTGTGAAGCACCTTTC |
| | MPRIP enhancer 1 | CACACACATATTCACAAGTCAGAGA | CATGCATGTTGGCCTGTTTATCT |
| Genotyping | N-term GFP-FKBP12-KLF5 knockin | TCTGAGGAGTCCACCCGAA | TCATGCTCAGCACCCTTGTA |

| siRNA | |
|---|---|
| Target | Sequence |
| KLF5 | GAUGUGAAAUGGAGAAGUA |
| KLF5 | CAACCUGUCAGAUACAAUA |
| KLF5 | UAUACCAAGUCUUCUCAUU |
| KLF5 | CCAGAGACCGUGCGUAACA |
| NT5 | UGGUUUACAUGUCGACUAA |

| sgRNA | |
|---|---|
| Target | Sequence |
| KLF5_Knockin_1 | TTGTAGCCATGGGCACTCGG |
| KLF5_Knockin_2 | GCTCAGCACCCTTGTAGCCA |
| BCL2L1_Enhancer_110kb_Left | ATTCCCAGGCCTCCCGCCCG |
| BCL2L1_Enhancer_110kb_Middle | GGCGCGCTGTTTGTCTAGCG |
| BCL2L1_Enhancer_110kb_Right | CAGGCGCCACTTTCCAGGAA |
| BCL2L1_Enhancer_130kb_Left | GGGCGCCAGGAACCGCCCCA |
| BCL2L1_Enhancer_130kb_Right | CTGAGACATTCAACCTCAGC |
| BCL2L1_Enhancer_160kb_Left | AGTCACGTAATGGCAATGCC |
| BCL2L1_Enhancer_160kb_Middle | GCTATTTCTAGGTCCCTGGC |
| BCL2L1_Enhancer_160kb_Right | TGGTATCAAATGGCTATCAG |

| Application | Target | Company | Catalog No. | Dilution/Amount | Opal | Notes |
|---|---|---|---|---|---|---|
| Western blot | KLF5 | Abcam | ab137676 | 1:1000 | | |
| | Bcl-xL | Cell Signaling | 2764 | 1:1000 | | |
| | HSPA8 | Cell Signaling | 8444 | 1:10000 | | |
| ChIP | KLF5 | Abcam | ab137676 | 5 µg | | |
| | H3K27ac | Diagenode | C15410196 | 1 µg | | |
| | H3K4me3 | Diagenode | C15410003 | 1 µg | | |
| | CTCF | Diagenode | C15410210 | 2 µg | | |
| Mulitplex immunofluorescence | KRT5/6 | Sigma Aldrich | MAB1620 | 1:25 | 480 | Panel #1 on patient tissue (Fig. 4) |
| | Bcl-xL | Cell Signaling | 2762 | 1:150 | 520 | |
| | p40 (ΔNp63) | Zeta Corporation | Z2004RL | 1:200 | 570 | |
| | HNF4α | Cell Signaling | 3113 | 1:100 | 620 | |
| | KLF5 | Abcam | ab137676 | 1:2000 | 690 | |
| | Pan-Keratin | Cell Signaling | 67306 | 1:50 | 780 | |
| | Ki-67 | Dako | M7240 | 1:100 | 480 | Panel #2 on knockin L3.6pl cells injected into mice (Fig. 6) |
| | αSMA | Abcam | ab5964 | 1:100 | 520 | |
| | Bcl-xL | Cell Signaling | 2764 | 1:100 | 570 | |
| | Cleaved caspase 3 | Cell Signaling | 9661 | 1:100 | 620 | |
| | KLF5 | Abcam | ab137676 | 1:100 | 690 | |
| | Cytokeratin 7 | Cell Marque | 307M | 1:25 | 780 | |

## Supplemental Material Western Blot Images Part 1

Fig. 2B

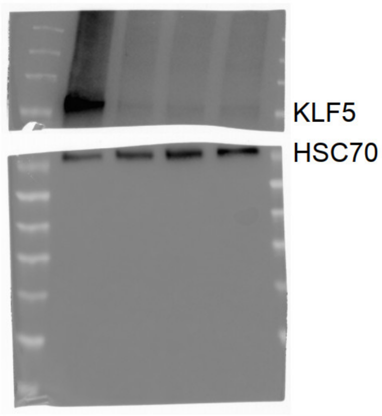


Fig. 2J

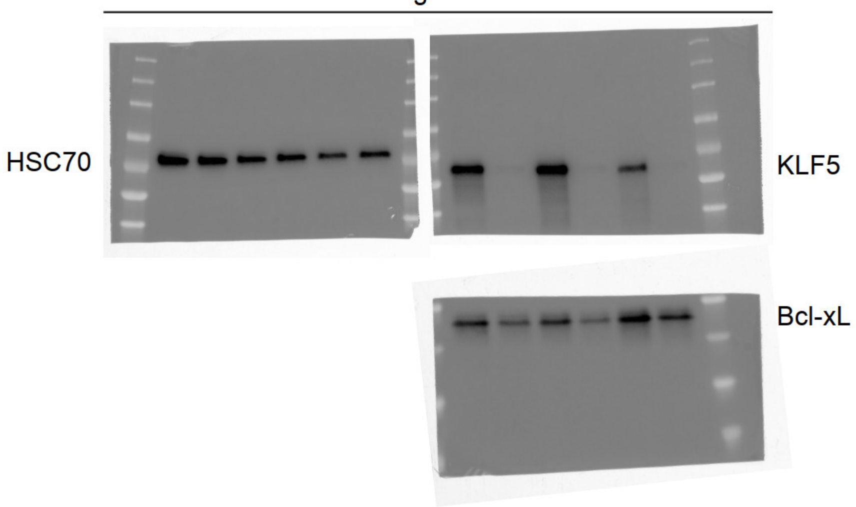


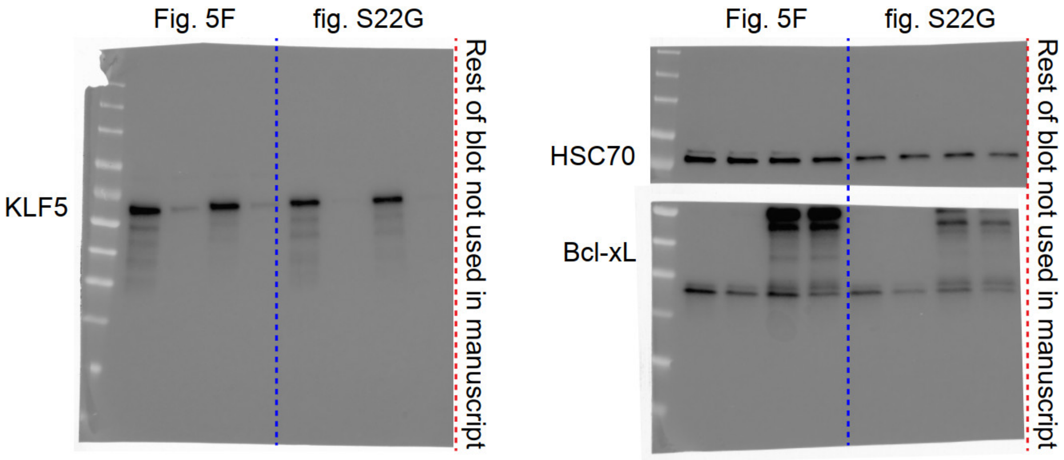


fig. S6A

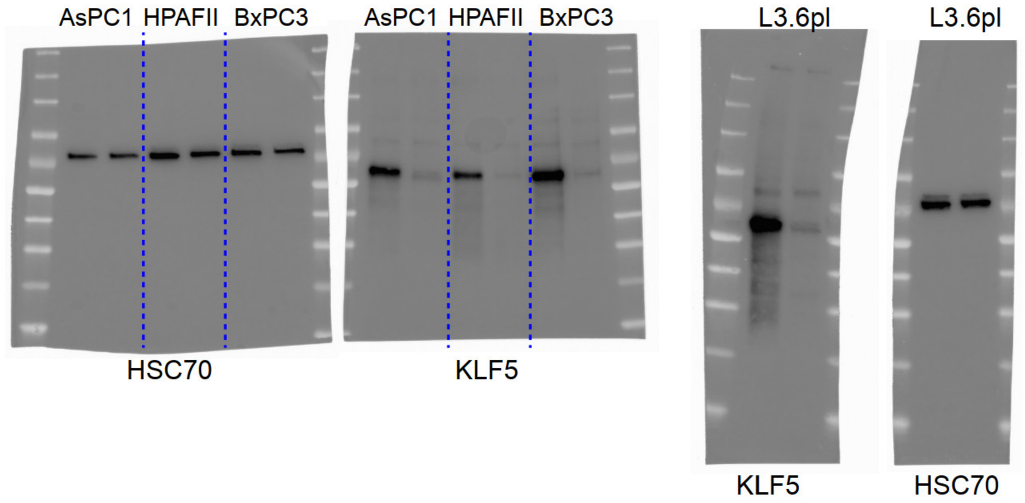

## Supplemental Material Western Blot Images Part 2

fig. S9C

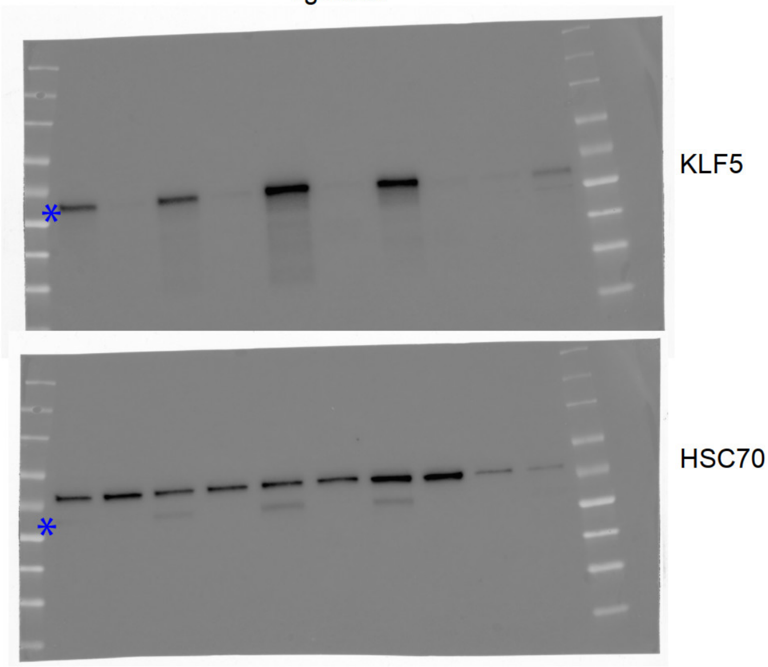


* incomplete ab stripping

fig. S10C

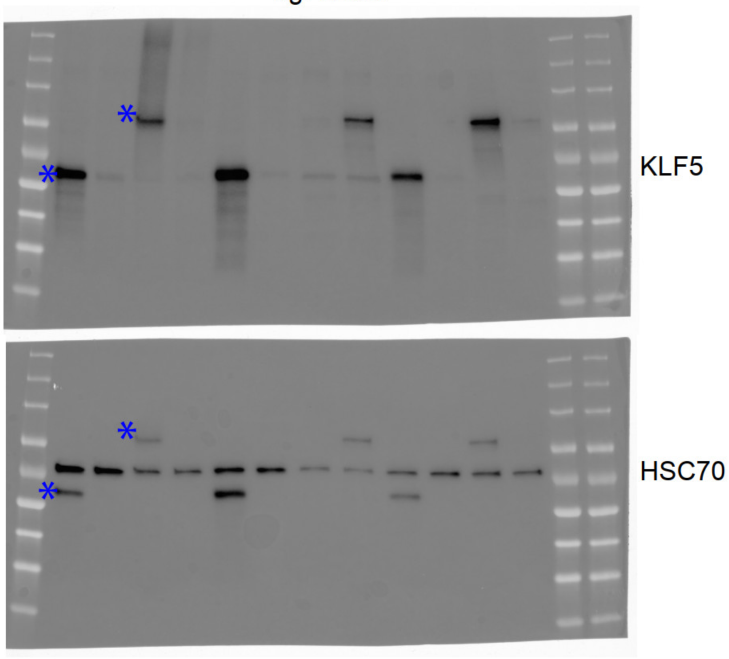


* incomplete ab stripping

fig. S17B

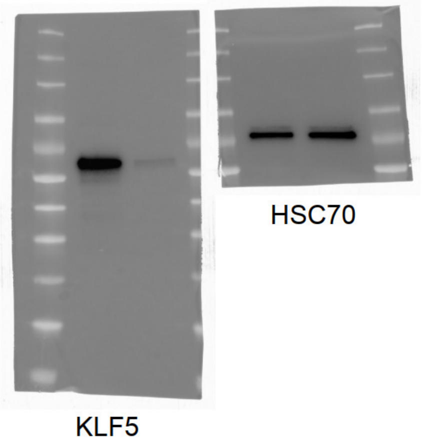


fig. S22E

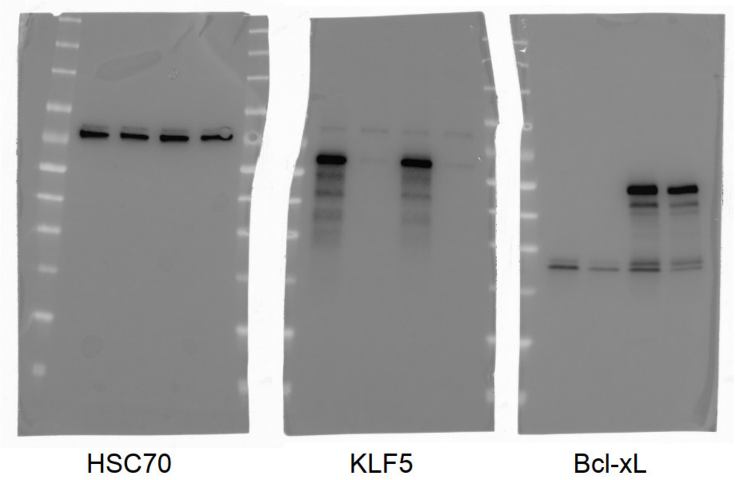

Supplement: Supplementary file 1 — Figs. S1 to S25 Tables S1 and S2 Western blot images [file sciadv.aea2106_sm.pdf]
